# Hybrid modeling approaches for agricultural commodity prices using CEEMDAN and time delay neural networks

**DOI:** 10.1038/s41598-024-74503-4

**Published:** 2024-11-04

**Authors:** Pramit Pandit, Atish Sagar, Bikramjeet Ghose, Moumita Paul, Ozgur Kisi, Dinesh Kumar Vishwakarma, Lamjed Mansour, Krishna Kumar Yadav

**Affiliations:** 1 Department of Agricultural Statistics & Computer Application, Rabindra Nath Tagore Agriculture College, Birsa Agricultural University, Ranchi, 834006 India; 2 Department of Agricultural Engineering, Rabindra Nath Tagore Agriculture College, Birsa Agricultural University, Ranchi, 834006 India; 3 Department of Agricultural Statistics, Bidhan Chandra Krishi Viswavidyalaya, Mohanpur, 741252 India; 4 Department of Civil Engineering, University of Applied Sciences, 23562 Lübeck, Germany; 5 Department of Civil Engineering, Ilia State University, Tbilisi, 0162 Georgia; 6 School of Civil, Environmental and Architectural Engineering, Korea University, Seoul, 02841 South Korea; 7 Department of Irrigation and Drainage Engineering, G. B. Pant University of Agriculture and Technology, Pantnagar, Uttarakhand, 263145 India; 8 Department of Zoology, College of Science, King Saud University, Riyadh, 11472 Saudi Arabia; 9 Department of Environmental Science, Parul Institute of Applied Sciences, Parul University, Vadodara, 391760 Gujarat India; 10 Environmental and Atmospheric Sciences Research Group, Scientific Research Center, Al-Ayen University, Thi- Qar, Nasiriyah, 64001 Iraq

**Keywords:** Agriculture price forecasting, Empirical mode decomposition, Intrinsic mode functions, Non-linearity, Time delay neural network, Applied mathematics, Software

## Abstract

Improving the forecasting accuracy of agricultural commodity prices is critical for many stakeholders namely, farmers, traders, exporters, governments, and all other partners in the price channel, to evade risks and enable appropriate policy interventions. However, the traditional mono-scale smoothing techniques often fail to capture the non-stationary and non-linear features due to their multifarious structure. This study has proposed a CEEMDAN (Complete Ensemble Empirical Mode Decomposition with Adaptive Noise)-TDNN (Time Delay Neural Network) model for forecasting non-linear, non-stationary agricultural price series. This study has evaluated its suitability in comparison with the other three major EMD (Empirical Mode Decomposition) variants (EMD, Ensemble EMD and Complementary Ensemble EMD) and the benchmark (Autoregressive Integrated Moving Average, Non-linear Support Vector Regression, Gradient Boosting Machine, Random Forest and TDNN) models using monthly wholesale prices of major oilseed crops in India. Outcomes from this investigation reflect that the CEEMDAN-TDNN hybrid models have outperformed all other forecasting models on the basis of evaluation metrics under consideration. For the proposed model, an average improvement of RMSE (Root Mean Square Error), Relative RMSE and MAPE (Mean Absolute Percentage Error) values has been observed to be 20.04%, 19.94% and 27.80%, respectively over the other EMD variant-based counterparts and 57.66%, 48.37% and 62.37%, respectively over the other benchmark stochastic and machine learning models. The CEEMD-TDNN and CEEMDAN-TDNN models have demonstrated superior performance in predicting the directional changes of monthly price series compared to other models. Additionally, the accuracy of forecasts generated by all models has been assessed using the Diebold-Mariano test, the Friedman test, and the Taylor diagram. The results confirm that the proposed hybrid model has outperformed the alternative models, providing a distinct advantage.

**Supplementary Information:**

The online version contains supplementary material available at 10.1038/s41598-024-74503-4.

## Introduction

The ever-increasing populace exerts considerable pressure on the agricultural production system while taking a simultaneous toll on fixed resources such as land and water. As a result, an increasing concern has been evident, and the associated uncertainty is expected to imply upward pressure on prices, especially in developing economies like India^1^. Moreover, the domestic and international market forces have contributed substantially to the increased price variability, and accorded importance to reliable and timely price forecasts^2–4^. These forecasts are expected to be useful for many stakeholders namely farmers, traders, exporters, governments, and all other partners in the price channel^4,5^. However, due to its strong dependence on biological processes and unpredictable natural events such as droughts, floods, pest, disease outbreaks, etc., agriculture price forecasting is considered a daunting task in the domain of time series forecasting^6–14^.

To strengthen this study’s foundation, recent literature highlights the significant potential and versatility of various machine learning models in modeling complex patterns across diverse research fields^2,9,15–26^. Among the numerous stochastic processes implemented, ARIMA (autoregressive integrated moving average) and its component models have virtually dominated agricultural commodity price forecasting^27^. However, these models assume a linear correlation structure among the time series observations. Therefore, if the observed feature of a real-world price series exhibits non-linearity, the forceful application of the ARIMA model is likely to result in unreliable forecasts and misleading economic implications. Several non-linear time series models, such as the bilinear model^28^and the threshold autoregressive (TAR) model^29^, were developed as early responses to overcome this impediment. However, these non-linear models also require a prespecified non-linear relationship, which may not be flexible enough to incorporate all the essential features^30–32^.

Different machine learning approaches, specifically artificial neural networks (ANNs)^6–8^, have emerged as viable alternatives to traditional predictive models^32–34^. Its non-parametric, data-driven, and self-adaptive nature has made it more appealing than other non-linear alternatives^35,36^. A comprehensive review of the literature on time series forecasting highlights the widespread interest among researchers in exploring the capabilities of artificial neural networks compared to traditional linear and non-linear statistical models^6–8^. In the context of agricultural price forecasting, Manogna and Mishra^5 ^illustrated the superior forecasting capabilities of neural network models in forecasting spot prices for agricultural commodities in India. Singh and Mishra^37 ^analyzed the groundnut oil price series in Mumbai and found that the ANN performed better than the ARIMA model in terms of mean squared error (MSE), root mean square error (RMSE), and mean absolute percentage error (MAPE) values. Areef and Radha^38^ compared the performance of ANN and generalized autoregressive conditional heteroskedastic (GARCH) models for forecasting potato prices in the Bengaluru market, Karnataka. It was apparent from their study that the forecasted prices were much closer to actual prices in the case of ANN rather than GARCH.

Even with the immense popularity and sheer power of the neural network models, their inability to model non-linear, non-stationary time series data has also been reported^39,40^. As a result, conventional mono-scale smoothing methods frequently struggle to accurately capture the intricate patterns and unpredictable fluctuations of agricultural commodity prices influenced by various factors^41–47^. As a result, it is logical to consider employing decomposition techniques to model price series, as they can address non-linearity and non-stationarity inherent in time series data^48–51^. Empirical mode decomposition (EMD) has demonstrated impressive competence in extracting valuable features from time series data characterized by non-linear and non-stationary attributes^51^. In contrast to conventional time series modeling, this self-adaptive decomposition method focuses on breaking down the original time series into multiple independent intrinsic mode functions (IMFs) along with a residue with varying amplitudes and frequencies. Consequently, recent years have witnessed the development and utilization of different EMD variations across divergent domains^52,53^. Table 1 provides an extensive review of the recent literature comparing different competitive decomposition strategies based on EMD in time series forecasting.

**Table 1:** Recent studies comparing different EMD-based techniques in time series forecasting.

Authors	Year	Salient outcomes
Niu et al.^54^	2016	Outperformance of the proposed CEEMD (Complementary EEMD)-based hybrid model over the EMD- and EEMD-based counterparts for short-term forecasting of $\:{\text{P}\text{M}}_{2.5}$ concentrations.
Fang et al.^55^	2018	Successful extraction of bearing fault characteristics for different fault signals by EEMD (Ensemble EMD) despite the failure of EMD.
Tayyab et al.^56^	2018	Clear advantages of EEMD over the discrete wavelet transform (DWT) for ANN-based prediction of streamflow at the upper Indus basin, Pakistan.
Aamir et al.^57^	2018	Superiority of CEEMDAN (Complete EEMD with Adaptive Noise)-ARIMA-Kalman filter combined model over EMD-based counterparts for crude oil price forecasting.
Cao et al.^58^	2019	Clear advantages of CEEMDAN over EMD for stock index price forecasting.
Fang et al.^59^	2020	Better forecasts of the EEMD combined model compared to individual SVR (Support Vector Regression), ANN, and ARIMA models in agricultural commodity future prices forecasting.
Lin et al.^60^	2021	Superiority of CEEMDAN as compared to EMD in forecasting stock index price.
Seyrek et al.^61^	2022	Superiority of the EEMD over EMD for intelligent chatter detection.
Liu et al.^62^	2022	Effectiveness of the EEMD algorithm for improving the prediction accuracy of the individual models in predicting the hourly urban water consumption.
Dhifaoui et al.^39^	2023	Better forecasts by combining the variable-lag transfer entropy framework and the EMD over the traditional variable-lag transfer entropy in detecting the causal interplays at various business cycles.
Liao et al.^63^	2023	Superiority of the EEMD-ANN model as compared to the ANN model for runoff forecasting.
Ahmad et al.^40^	2024	Outperformance of the EMD-ANN model over A.I. (Artificial Intelligence)-based models in water resource management and flood mitigation.
Shahbazi et al.^64^	2024	Better forecasts of the hybrid CEEMD-ANN model compared to the wavelet transform (WT) in combination with ANN and SVR in predicting the groundwater level in aquifers.
Zhang et al.^53^	2024	Improved prediction accuracy and adaptability of the constructed CEEMDAN–based hybrid model over its EMD–based and individual counterparts in forecasting monthly average temperature in Jinan City, Shandong Province.

At this juncture, it is important to note that the forecasting of agricultural commodities prices differs from typical time series data due to its unique characteristics. However, few studies have been conducted on decomposition-based agricultural commodity price forecasting. There needs to be up to date and proper state of the art regarding the theoretical foundation and practical applications of decomposition-based modeling for addressing non-stationarity and non-linearity in agricultural price forecasting. This accounts for a research gap arising from the need for an in-depth understanding of effectively implementing and integrating decomposition techniques with advanced models and correctly interpreting and analyzing the results. Further, considering agricultural price forecasting as a sub-domain of time series analysis, the significance of each step in the implementation process is essential for understanding the statistical implications, an aspect often overlooked in existing studies. For example, most of the studies^40,53,59,64,65^ did not consider the essential steps of statistics, such as pretesting for non-stationarity and non-linearity, while applying EMD-based decompositions or non-linear A.I. (Artificial Intelligence) models for forecasting. Furthermore, despite introducing advanced versions of EMD over time, a comprehensive empirical comparison of these variants in the context of agricultural price systems needs to be more present. These observations underscore the need for a systematic examination in forecasting agricultural price series using decomposition-based hybrid models.

Hence, in this paper, encouraged by the successful application of CEEMDAN-based hybrid models across various fields and with the aim of filling the identified research gap, we propose a CEEMDAN-TDNN hybrid model. This model is designed to effectively capture the non-stationary and non-linear characteristics of agricultural price series data. We empirically evaluate the performance of our proposed model in forecasting the monthly wholesale prices of major oilseed crops in India, comparing it to three other prominent EMD variants (EMD, EEMD, and CEEMD). Three additional A.I. models, including Non-linear Support Vector Regression (NLSVR), Gradient Boosting Machine (GBM), and Random Forest (RF), which have gained upsurging interest in the field of artificial intelligence and predictive modeling^10,12,66–72^, have also been included in the study alongside ARIMA and TDNN as benchmark models for comparative analysis. The most effective model is determined based on performance evaluation metrics. To ensure robust validation and assess the forecasting superiority of the developed model, both parametric (Diebold-Mariano test) and non-parametric (Friedman test) tests, as well as a graphical approach (Taylor diagram), have been utilized. Moreover, accurately forecasting the price direction for the upcoming month is deemed a crucial factor in model selection. These forecasts are particularly valuable in economics for understanding the phases of the business cycle, specifically about turning points.

The oilseed price series in this study were chosen purposefully keeping the nature of the data required and the economic importance of these markets in mind^73,74^. The remainder of this paper proceeds as follows. ‘Materials and Methods’ provides the data used for the experimentation and elaborates on the time series forecasting methods adopted in this paper. Empirical results from real data and the relevant discussion are provided in ‘Results’ and ‘Discussion’, respectively. The last section eventually concludes.

## Materials and methods

### Data

For the current investigation on monthly wholesale price (₹/q) of significant oilseed crops, data series from January 2008 to December 2019 are obtained from the various issues of ‘Agricultural Prices in India’ published by the Directorate of Economics & Statistics, Department of Agriculture and Farmers Welfare, Ministry of Agriculture and Farmers Welfare, Government of India. Three markets, namely Rajkot, Delhi, and Kanpur have been considered for Groundnut, rapeseed & mustard, and linseed, respectively. For all the markets, out of the 144 observations available, the first 132 observations are utilized for model building while retaining the rest for testing.

### Time series forecasting methods

#### The ARIMA model

ARIMA models are an extension of ARMA models that use an appropriate order of differencing to handle a wide range of non-stationary time series^75,76^. ARIMA assumes that the differenced series is a linear function of past actual values and random shocks^75^. A process $\:\left\{{\text{y}}_{\text{t}}\right\}$ is said to follow an ARIMA (p, d, q) model if it can be expressed as:

1 $$\:{\phi\:}\left(\text{B}\right){\left(1-\text{B}\right)}^{\text{d}}{\text{y}}_{\text{t}}={\uptheta\:}\left(\text{B}\right){{\upepsilon\:}}_{\text{t}}$$

or

2 $$\:\left(1-{{\phi\:}}_{1}\text{B}-{{\phi\:}}_{2}{\text{B}}^{2}-\dots\:-{{\phi\:}}_{\text{p}}{\text{B}}^{\text{p}}\right){\left(1-\text{B}\right)}^{\text{d}}{\text{y}}_{\text{t}}=\left(1-{{\uptheta\:}}_{1}\text{B}-{{\uptheta\:}}_{2}{\text{B}}^{2}-\dots\:-{{\uptheta\:}}_{\text{q}}{\text{B}}^{\text{q}}\right){{\upepsilon\:}}_{\text{t}}$$

where p, d, and q, being non-negative integers, refer to the order of autoregression, differencing, and moving average, respectively. B is the backshift operator defined as $\:\text{B}{\text{y}}_{\text{t}}={\text{y}}_{\text{t}-1}$. $\:{\phi\:}\left(\text{B}\right)$ and $\:{\uptheta\:}\left(\text{B}\right)$ are respective polynomials of degree p and q in B. The random error $\:{{\upepsilon\:}}_{\text{t}}$ is supposed to be a standard white noise process following $\:\text{N}(0,{{\upsigma\:}}^{2})$. A detailed discussion of various aspects of this method can be found in Box et al.^77^.

#### The TDNN model

ANNs are a class of non-linear, non-parametric, self-adaptive, and data-driven computational methods^30,32,78^. These are specifically useful when the underlying data relationship is unknown. A general neural network architecture consists of an input layer that receives the input data, one or more hidden layers that offer non-linearity to the model, and an output layer that yields the target value^79^. The general expression for a multilayer feed-forward neural network is represented by:

3 $$\:{y}_{t}={\alpha\:}_{0}+\sum\:_{j=1}^{q}{\alpha\:}_{j}g\left({\beta\:}_{0j}+\sum\:_{i=1}^{p}{\beta\:}_{ij}{y}_{t-i}\right)+{\epsilon\:}_{t}$$

where $\:{{\upalpha\:}}_{\text{j}}\:(\text{j}=\text{0,1},2,\dots\:,\text{q})$ and $\:{{\upbeta\:}}_{\text{i}\text{j}}\:(\text{i}=\text{0,1},2,\dots\:,\text{p})$ are the model parameters, often called as the connection weights. p and q refer to the number of input and hidden nodes, respectively.

ANN can represent time series data by offering an implicit functional representation of time, whereby a static neural network, such as a multilayer perceptron, is assigned dynamic properties. One of the easiest ways to embed short-term memory into a neural network’s structure is to utilize time delay at the input layer. One such architecture is TDNN. The logistic function has served as the hidden layer activation function with the form:

4 $$\:\text{f}\left(y\right)=\frac{1}{1+{\text{e}}^{-\text{y}}}$$

A typical TDNN structure with one hidden layer is denoted by I: Hs: O, where I, H and O are the number of nodes in the input, hidden layer, and output layer, respectively and s denotes the logistic transfer function. For p input nodes (tapped delay), q hidden nodes, one output node, and biases at both hidden and output layers, the total number of weights in a three-layer feed-forward neural network is q (p + 2) + 1.

#### The NLSVR model

In the context of a given data set {$\:{\left\{{\text{x}}_{\text{i}},{\text{y}}_{\text{i}}\:\right\}}_{\text{i}=1}^{\text{n}}$, with $\:{\text{x}}_{\text{i}}\in\:{\text{R}}^{\text{n}}$ representing the input vector, $\:{\text{y}}_{\text{i}}\in\:\text{R}$ as the scalar output, and n denoting the size of the data set, the NLSVR estimating function^80^ can be expressed in the general form as follows:

5 $$\:\text{f}\left(\text{x}\right)={\text{w}}^{\text{T}}{\phi\:}\left(\text{x}\right)+\text{b}$$

Here, $\:{\phi\:}\left(.\right)$ denotes a non-linear mapping function that transforms the original input space into a higher dimensional feature space. In this equation, w represents the weight vector, b denotes the bias term, and the superscript T signifies the transpose operation. The coefficients w and b are estimated from data by minimizing the following regularized risk function:

6 $$\:\text{R}\left({\uptheta\:}\right)=\frac{1}{2}{\Vert\text{w}\Vert}^{2}+\text{C}\left[\frac{1}{\text{n}}\sum\:_{\text{i}=1}^{\text{n}}{\text{L}}_{{\upepsilon\:}}({\text{y}}_{\text{i}},\:\text{f}({\text{x}}_{\text{i}}\left)\right)\right]$$

In the equation above, the term $\:\frac{1}{2}{\Vert\text{w}\Vert}^{2}$ is referred to as the ‘regularized term’, which evaluates the smoothness of the function. The term $\:\frac{1}{\text{n}}\sum\:_{\text{i}=1}^{\text{n}}{\text{L}}_{{\upepsilon\:}}({\text{y}}_{\text{i}},\:\text{f}({\text{x}}_{\text{i}}\left)\right)$ is known as the ‘empirical error’, and it is estimated using the Vapnik ε-insensitive loss function. Both C and ε are hyperparameters that the user can set. The Vapnik Loss function is defined as:

7 $$\:{\text{L}}_{{\upepsilon\:}}\left({\text{y}}_{\text{i}},\:\text{f}\left({\text{x}}_{\text{i}}\right)\right)=\{\begin{array}{c}\left|{\text{y}}_{\text{i}}-\:\text{f}\left({\text{x}}_{\text{i}}\right)\right|-{\upepsilon\:}\:\:\:\:\:\:\text{w}\text{h}\text{e}\text{n}\:\left|{\text{y}}_{\text{i}}-\:\text{f}\left({\text{x}}_{\text{i}}\right)\right|\ge\:{\upepsilon\:}\\\:\:0\:\:\:\:\:\:\:\:\text{w}\text{h}\text{e}\text{n}\:\:\:\:\:\:\:\:\:\left|{\text{y}}_{\text{i}}-\:\text{f}\left({\text{x}}_{\text{i}}\right)\right|<{\upepsilon\:}\end{array}$$

where $\:{\text{y}}_{\text{i}}$ represents the actual value and $\:\text{f}\left({\text{x}}_{\text{i}}\right)$ indicates the estimated value at $\:{\text{i}}^{\text{t}\text{h}}$ period.

#### The GBM model

Gradient Boosting^66^ is a powerful boosting algorithm that merges multiple weak learners to form strong learners. Each successive model is trained using gradient descent to reduce the loss function, like the mean squared error, of the preceding model. During each iteration, the algorithm calculates the gradient of the loss function concerning the current ensemble’s predictions. Subsequently, a new weak model is trained to minimise this gradient. The new model’s predictions are then incorporated into the ensemble, and the process continues until a particular stopping criterion is satisfied.

Let us consider an ensemble comprising M trees. Tree-1 is trained with feature matrix X and output vector y. The predictions $\:{\widehat{\text{y}}}_{1}$ are used to calculate residuals $\:{\text{r}}_{1}$. Tree-2 is subsequently trained using feature matrix X as input and the residual error $\:{\text{r}}_{1}$ from Tree-1 as the output. The predicted results $\:{\widehat{\text{r}}}_{1}$ are then used to calculate residual $\:{\text{r}}_{2}$. This process is iterated until all M trees in the ensemble are trained. A critical parameter utilized in this technique is ‘Shrinkage’, which involves reducing the prediction of each tree in the ensemble by multiplying it with the learning rate (η) that falls within the range of 0 to 1. A trade-off exists between η and the number of estimators, as reducing the learning rate requires compensation with an increase in estimators to achieve a specific model performance level. Once all trees are trained, predictions can be generated. Each tree predicts an output vector, and the final prediction $\:\widehat{\text{y}}$ can be calculated using the following formula:

8 $$\:\widehat{\text{y}}={\widehat{\text{y}}}_{1}+\left({\upeta\:}\times\:{\widehat{\text{r}}}_{1}\right)+\left({\upeta\:}\times\:{\widehat{\text{r}}}_{2}\right)+\dots\:+\left({\upeta\:}\times\:{\widehat{\text{r}}}_{\text{M}-1}\right)$$

#### The random forest model

Bagging or bootstrap aggregation decreases the variance of an estimated prediction function^65,81^. It is particularly effective for procedures with high variance and low bias, like trees. In the regression case, the same regression tree is fitted to bootstrapped training data samples, and the outcomes are averaged. Random forests represent a significant adaptation of bagging, constructing a vast array of uncorrelated trees and averaging them^71^. The algorithm for regression with random forests can be summarized as follows:

i. Generate a bootstrap sample of size N from the training dataset.
ii. Construct a random forest tree $\:{\text{T}}_{\text{b}}$ (b = 1, 2, …, B) using the bootstrapped data by iteratively applying the following steps (a)-(c) up to each terminal node of the tree until the minimum node size $\:{\text{n}}_{\text{m}\text{i}\text{n}}$ is reached:

- (a) Pick m variables at random from the total p variables.
- (b) Determine the best variable/split-point from the selected m variables.
- (c) Divide the node into two daughter nodes.

iii. Combine the output of all trees {$\:{\left\{{\text{T}}_{\text{b}}\right\}}_{\text{b}=1}^{\text{B}}$ to obtain the final prediction.
iv. Make predictions for a new data point x using the random forest model:

9 $$\:{\widehat{f}}_{RF}^{B}\left(x\right)=\frac{1}{B}\sum\:_{b=1}^{B}{T}_{b}\left(x\right)$$

#### EMD

The EMD decomposes the complex original series into a series of IMFs and a residue based on the local characteristics of the series such as the local maxima, local minima, and zero-crossings^60^. Its essence lies in transitioning from non-stationary and non-linear signals to linear and stationary ones. Since the features are obtained empirically, the process is adaptive and efficient. The EMD method can be depicted as follows^82^.

i. Identify all local maxima and local minima of the original time series y(t).
ii. Obtain an upper envelope u(t) and a lower envelope l(t) by interpolating all the local extrema.
iii. Calculate the average of the upper and lower envelopes as $\:\text{m}\left(\text{t}\right)=\frac{\text{u}\left(\text{t}\right)+\text{l}\left(\text{t}\right)}{2}$.
iv. Obtain a detailed component d(t) by subtracting the average m(t) from the original time series y(t) as $\:d\left(t\right)=y\left(t\right)-m\left(t\right)$.
v. If m(t) and d(t) meet any stopping criteria, then the first IMF $\:{\text{c}}_{1}\left(\text{t}\right)=\text{m}\left(\text{t}\right)$ and the first residue $\:{\text{r}}_{1}\left(\text{t}\right)=\text{d}\left(\text{t}\right).$ The stopping criterion refers to if m(t) tends to zero or the number of local extrema and the number of zero crossings of d(t) maximally differs by one or the user-defined maximum iteration is reached. Rilling et al.^36^ have also reported two threshold-based stopping criteria specified as:

10 $$\:\frac{c(t\left|\delta\:\left(t\right)<{\theta\:}_{1})\right.}{c\left(t\right)}\ge\:1-\alpha\:$$

11 $$\:{\updelta\:}\left(\text{t}\right)<{{\uptheta\:}}_{2}$$

where $\:{\updelta\:}\left(\text{t}\right)=\left|\frac{\text{u}\left(\text{t}\right)+\text{l}\left(\text{t}\right)}{\text{u}\left(\text{t}\right)-\text{l}\left(\text{t}\right)}\right|$ and α, $\:{{\uptheta\:}}_{1}$, $\:{{\uptheta\:}}_{2}$ are user-defined constants. c(.) denotes a function to count the numbers in a set.

vi. However, if none of the stopping criteria are reached after step (iv), repeat steps (i) to (iv) until all (let n) the IMFs and the residue are obtained.
vii. Finally, the original time series is reconstructed as:

12 $$\:y\left(t\right)=\sum\:_{j=1}^{n}{c}_{j}\left(t\right)+{r}_{n}\left(t\right)$$

#### EEMD

The major lacuna of EMD application is the problem of mode mixing, in which an IMF is made up of signals covering a large frequency range, or many IMFs comprising signals in a similar frequency band exist^83^. To tackle this problem, Wu and Huang^84 ^developed an ensemble version of EMD called EEMD. In EEMD, multiple trials are carried out and each trial is similar to EMD except that the input series is a mixture of the original time series at hand and a finite Gaussian white noise. Although the resulting decompositions are noisier, the uncorrelated finite white noise will negate each other in the time of mean computation over all trials. Thus, the relevant time series can be retained, eliminating the mode mixing problem. The EEMD procedure is as follows^84^:

i. Generate several noise-added time series by adding independent Gaussian white noises.

13 $$\:{y}^{i}\left(t\right)=y\left(t\right)+{\epsilon\:}^{i}\left(t\right)$$

where i = 1, 2,…, I. $\:{\text{y}}^{\text{i}}\left(\text{t}\right)$ and $\:{{\upepsilon\:}}^{\text{i}}\left(\text{t}\right)$ denote the $\:{\text{i}}^{\text{t}\text{h}}$ noise-added series and $\:{\text{i}}^{\text{t}\text{h}}$ independent Gaussian white noise, respectively.

ii. For each $\:{\text{y}}^{\text{i}}\left(\text{t}\right)$, EMD is applied to obtain the decomposed IMFs and residue as:

14 $$\:{y}^{i}\left(t\right)=\sum\:_{j=1}^{n}{c}_{j}^{i}\left(t\right)+{r}_{n}^{i}\left(t\right)$$

iii. The original time series can be reconstructed by averaging over all trials.

15 $$\:y\left(t\right)=\frac{1}{I}\left(\sum\:_{i=1}^{I}\sum\:_{j=1}^{n}{c}_{j}^{i}\left(t\right)+{r}_{n}^{i}\left(t\right)\right)+{\epsilon\:}_{I}\left(t\right)$$

where $$\:{{\upepsilon\:}}_{\text{I}}\left(\text{t}\right)=\frac{{\upepsilon\:}\left(\text{t}\right)}{\sqrt{\text{I}}}$$.

#### CEEMD

Even though EEMD is a remarkable improvement over EMD in terms of stability, it cannot still completely neutralize the added noise. Hence, Yeh et al.^85^ developed CEEMD, which can achieve the same decomposition effect as EEMD while reducing the reconstruction error by using paired noise with positive and negative signals. The CEEMD procedure is the same as EEMD except $$\:{{\upepsilon\:}}^{\text{i}}\left(\text{t}\right)\in\:\left\{{{\upepsilon\:}}_{+}^{\raisebox{1ex}{$\text{i}$}\!\left/\:\!\raisebox{-1ex}{$2$}\right.}\left(\text{t}\right),\:\:{{\upepsilon\:}}_{\_}^{\raisebox{1ex}{$\text{i}$}\!\left/\:\!\raisebox{-1ex}{$2$}\right.}\left(\text{t}\right)\right\}$$, where $$\:{{\upepsilon\:}}_{+}^{\raisebox{1ex}{$\text{i}$}\!\left/\:\!\raisebox{-1ex}{$2$}\right.}\left(\text{t}\right)+\:{{\upepsilon\:}}_{\_}^{\raisebox{1ex}{$\text{i}$}\!\left/\:\!\raisebox{-1ex}{$2$}\right.}\left(\text{t}\right)=0$$; i = 1, 2,…, I.

#### CEEMDAN

Another problem associated with EEMD is the high cost of computation. To minimize the number of trials while preserving the capability of solving the mode mixing problem, Torres et al.^86^ proposed CEEMDAN. The CEEMDAN method proceeds as follows.

i. Generate several noise-added time series by adding independent Gaussian white noises with unit variance.

16 $$\:{y}^{i}\left(t\right)=y\left(t\right)+{\omega\:}_{0}{\epsilon\:}^{i}\left(t\right)$$

where i = 1, 2,…, I and $\:{{\upomega\:}}_{0}$ denotes the noise coefficient.

ii. For each $\:{\text{y}}^{\text{i}}\left(\text{t}\right)$, apply EMD to obtain the first decomposed IMF and calculate the mean as $\:{\text{c}}_{1}\left(\text{t}\right)=\frac{1}{\text{I}}\sum\:_{\text{i}=1}^{\text{I}}{\text{c}}_{1}^{\text{i}}\left(\text{t}\right)$. Subsequently, the first residue is obtained as: $\:{\text{r}}_{1}\left(\text{t}\right)=\text{y}\left(\text{t}\right)-{\text{c}}_{1}\left(\text{t}\right)$.
iii. Decompose the noise-added residue to obtain the second IMF:

17 $$\:{\text{c}}_{2}\left(\text{t}\right)=\frac{1}{\text{I}}\sum\:_{\text{i}=1}^{\text{I}}{\text{E}}_{1}\left({\text{r}}_{1}\left(\text{t}\right)+{{\upomega\:}}_{1}{\text{E}}_{1}\left({{\upepsilon\:}}^{\text{i}}\left(\text{t}\right)\right)\right)$$

where $\:{\text{E}}_{\text{j}}(.)$ refers to a function to extract the $\:{\text{j}}^{\text{t}\text{h}}$ IMF decomposed by EMD.

iv. Repeat for the remaining IMFs until at most two extrema of the residue exist.

**Fig. 1:**
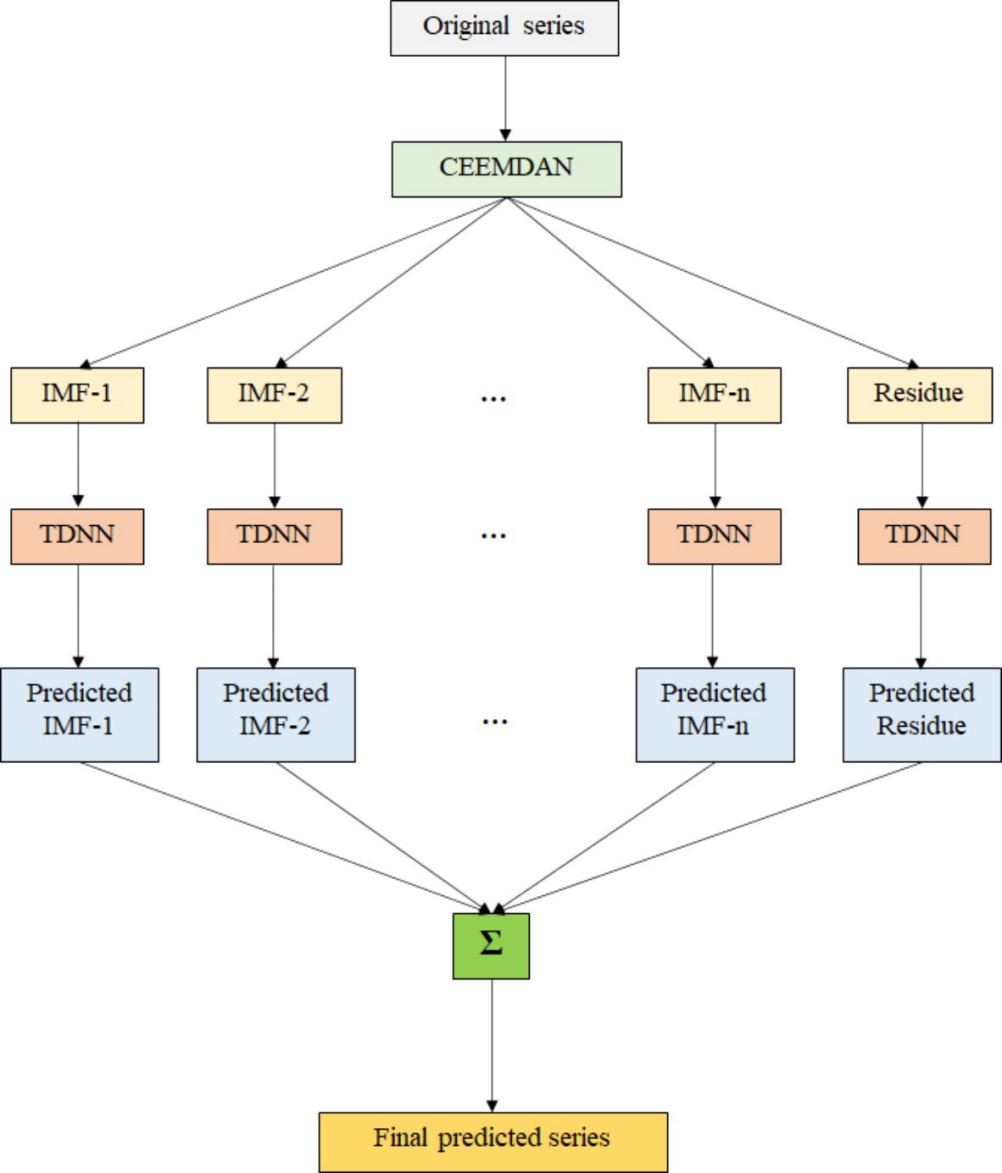
Schematic representation of the CEEMDAN-TDNN model.

The proposed CEEMDAN-TDNN hybrid modeling technique is schematically represented in Fig. 1.

### Evaluation of forecasting accuracy

#### Evaluation metrics

The forecasting performance of the models employed in this study is evaluated concerning three common accuracy measures, viz., RMSE, RRMSE (Relative RMSE) and MAPE. However, Niu and Xu^87^argue that the forecasting performance of non-linear models should be assessed by their ability to correctly predict the direction of change rather than by error-based measures such as RMSE, RRMSE, MAPE, etc. Hence, a comprehensive evaluation has been carried out in terms of both error-based measures (RMSE, RRMSE and MAPE)^88–92^ and the directional prediction statistics $\:\left({\text{D}}_{\text{s}\text{t}\text{a}\text{t}}\right)$^93–95^.

18 $$\:\text{R}\text{M}\text{S}\text{E}=\sqrt{\frac{1}{\text{n}}\sum\:_{\text{t}=1}^{\text{n}}{({\text{y}}_{\text{t}}-\widehat{{\text{y}}_{\text{t}}})}^{2}}$$

19 $$\:\text{M}\text{A}\text{P}\text{E}=\frac{1}{\text{n}}\sum\:_{\text{t}=1}^{\text{n}}\left|\frac{{\text{y}}_{\text{t}}-\widehat{{\text{y}}_{\text{t}}}}{{\text{y}}_{\text{t}}}\right|\times\:100\text{\%}$$

20 $$\:\text{R}\text{R}\text{M}\text{S}\text{E}=\frac{\sqrt{\frac{1}{\text{n}}\sum\:_{\text{t}=1}^{\text{n}}{\left({\text{y}}_{\text{t}}-\widehat{{\text{y}}_{\text{t}}}\right)}^{2}}}{\sum\:_{\text{t}=1}^{\text{n}}\widehat{{\text{y}}_{\text{t}}}}\times\:100$$

21 $$\:{\text{D}}_{\text{s}\text{t}\text{a}\text{t}}=\frac{1}{\text{n}}\sum\:_{\text{t}=1}^{\text{n}}{\text{a}}_{\text{t}}\times\:100\text{\%}$$

where $\:{\text{a}}_{\text{t}}=1$ if $\:({\text{y}}_{\text{t}}-{\text{y}}_{\text{t}-1})(\widehat{{\text{y}}_{\text{t}}}-{\widehat{\text{y}}}_{\text{t}-1})\ge\:0$, otherwise $\:=0$.

where $\:{\text{y}}_{\text{t}}$ and $\:\widehat{{\text{y}}_{\text{t}}}$ denote the $\:{\text{t}}^{\text{t}\text{h}}$ actual and predicted values in the test data set. n refers to the size of the test set.

#### Taylor diagram

Taylor diagrams^34,96^ provide a visual representation of the correspondence between patterns and observations. This comparison is based on measures of correlation, centered root-mean-square difference, and standard deviations. These diagrams are valuable for assessing the relative performance of various models. The Taylor diagram illustrates the statistical connection between two datasets: a ‘test field’ (typically a model simulation) and a ‘reference field’ (usually observational data). Each point on the diagram represents three distinct statistics simultaneously - the centered RMS difference, correlation, and standard deviation, due to their interrelatedness as defined by the formula below:

22 $$\:{\text{E}}^{{\prime\:}2}={{\upsigma\:}}_{\text{f}}^{2}+{{\upsigma\:}}_{\text{r}}^{2}-2{{\upsigma\:}}_{\text{f}}{{\upsigma\:}}_{\text{r}}\text{R}$$

where R is the correlation coefficient between the test and reference fields, E’ is the centred RMS difference between the fields, and $\:{{\upsigma\:}}_{\text{f}}^{2}$ and $\:{{\upsigma\:}}_{\text{r}}^{2}$ are the variances of the test and reference fields, respectively. The diagram is constructed based on the correlation given by the cosine of the azimuthal angle, drawing similarities between the equation and the Law of Cosines:

23 $$\:{\text{c}}^{2}={\text{a}}^{2}+{\text{b}}^{2}-2\text{a}\text{b}\text{c}\text{o}\text{s}{\phi\:}$$

#### Tests of significance for equal forecasting ability

To confirm the accuracy and evaluate the effectiveness of the developed forecasting model, we have conducted both the Diebold–Mariano test^97^and Friedman test^98^. Instructions for conducting these tests are detailed in ‘Supplementary Information S1’.

## Results

### Basic features

The basic features of the price series involved in this experiment are briefed in Table 2. The average price in these markets is around ₹ 3700–3900 per quintal. The CV(%) values indicate the presence of a relatively higher degree of instability in the data series. The auto-correlation and partial auto-correlation functions display no significant and regular seasonal pattern. Seasonal indices, as presented in Table 3, further confirm it. The augmented Dickey-Fuller (ADF) test^99^ has been utilized to decide on the non-seasonal differencing of the price series under study and is reported in Table 4. For all the markets, non-stationarity and stationarity have been observed for the level and first difference series, respectively.

**Table 2:** Descriptive statistics of the oilseed price series.

Price series	Groundnut	Rapeseed & mustard	Linseed
Mean	3702.89	3680.19	3893.45
Minimum	2120.00	2400.00	2225.00
Maximum	9690.00	5000.00	6530.00
Standard Deviation	973.44	650.83	940.03
CV (%)	26.29	17.68	24.14
Skewness	1.68	0.06	0.44
Kurtosis	8.51	1.15	0.18

As our investigation focuses on modeling techniques for non-linear, non-stationary time series data, assessing if the provided time series is non-linear before proceeding further is crucial. The Brock–Dechert–Scheinkman (BDS) test^100^ has been implemented in this study to test non-linearity. This test examines the spatial dependence of the observed series. The results in Table 5 reflect the strong rejection of linearity in all cases. Therefore, upon confirmation of these series’ non-linear and non-stationary nature, EMD and its improved variants can be effectively implemented for these price series forecasting.

**Table 3:** Seasonal indices of the oilseed price series.

Months	Groundnut	Rapeseed & mustard	Linseed
January	1.06	0.99	0.98
February	0.95	0.98	0.96
March	0.96	0.94	0.94
April	1.01	0.96	0.96
May	1.01	0.98	0.96
June	1.00	0.98	1.00
July	1.01	1.00	1.01
August	1.00	1.01	1.03
September	0.99	1.01	1.03
October	0.98	1.04	1.03
November	1.01	1.05	1.04
December	1.02	1.06	1.06

**Table 4:** Results of the ADF test.

Price series	Level series		First difference series	
	t-statistic	*p* value	t-statistic	*p* value
Groundnut	-2.68	0.29	-6.01	< 0.01
Rapeseed & mustard	-3.31	0.07	-4.84	< 0.01
Linseed	-2.61	0.32	-4.57	< 0.01

**Table 5:** Results of the BDS test.

Price series	Epsilon	Embedding dimension (m = 2)		Embedding dimension (m = 3)	
		Statistic	p value	Statistic	p value
Groundnut	303.59	6.90	< 0.01	7.92	< 0.01
	607.18	6.82	< 0.01	7.12	< 0.01
	910.77	6.11	< 0.01	6.11	< 0.01
	1214.36	7.30	< 0.01	6.95	< 0.01
Rapeseed & mustard	72.93	6.43	< 0.01	7.54	< 0.01
	145.87	5.25	< 0.01	5.45	< 0.01
	218.80	3.29	< 0.01	3.01	< 0.01
	291.74	1.27	0.20	1.02	0.31
Linseed	93.57	2.76	< 0.01	3.24	< 0.01
	187.14	3.88	< 0.01	4.41	< 0.01
	280.70	4.60	< 0.01	4.95	< 0.01
	374.27	4.66	< 0.01	4.75	< 0.01

### Implementation

The present study applies ARIMA, NLSVR, GBM, RF and TDNN models to the original series. For the EMD variant-based models, the IMFs and residue are obtained first and then the appropriate models are selected for each sub-series.

#### Fitting of the ARIMA models

The ACF and PACF plots serve as a reliable guide for the possible order of the ARIMA model. Minimum Akaike information criteria (AIC), Bayesian information criteria (BIC) and minimum RMSE, RRMSE and MAPE values have been used to select the best model. The parameter estimates of the selected ARIMA models are provided in Table 6.

**Table 6:** Parameter estimates of the fitted ARIMA models.

Price series	Parameter	Estimate	*p* value
Groundnut	C	11.62	0.76
	$\:{{\phi\:}}_{1}$	-0.41	< 0.01
Rapeseed & mustard	C	14.20	0.32
	$\:{{\uptheta\:}}_{1}$	0.10	0.09
Linseed	C	17.50	0.47
	$\:{{\uptheta\:}}_{1}$	0.23	< 0.01
	$\:{{\uptheta\:}}_{2}$	0.27	< 0.01

#### Fitting of the machine learning models

One crucial aspect of NLSVR modeling is the selection of hyper-parameters. The performance of NLSVR is significantly affected by the choice of input lags, kernel function, regularization parameter, kernel width, and margin of tolerance. For this study, we have utilized the popular radial basis function (RBF) as the kernel function to construct NLSVR models following the specifications provided in Table 7.

The performance of the GBM also crucially relies on the optimal selection of hyper-parameters. The hyper-parameters fine-tuned for training this model include the number of input lags, number of estimators, maximum depth, minimum samples per leaf, subsample, and learning rate. The best-performing GBM models have been developed according to the specifications outlined in Table 7.

Similarly, when tuning random forests, it is essential to consider the number of input lags, number of estimators, minimum samples per leaf, and maximum depth. Several automated techniques in existing literature can be utilized for hyper-parameter combinations. Among these methods, we have opted for grid search, which systematically explores all possible combinations of the hyper-parameters. The results of the optimized configurations can be found in Table 7.

**Table 7:** Specifications of the fitted machine learning models.

Specifications	Groundnut	Rapeseed & mustard	Linseed
**NLSVR**			
No. of input lags	2	2	4
C	10.00	20.00	1.00
γ	0.50	0.25	0.25
ε	0.25	0.10	0.10
Friedman F rank	3.33	3.00	3.00
**GBM**			
No. of input lags	4	2	1
No. of estimators	500	1000	200
Maximum depth	25	25	1
Minimum samples per leaf	20	20	5
Subsample	0.7	0.7	0.8
Learning rate	0.01	0.05	0.05
Friedman F rank	3.33	3.33	4.67
**RF**			
No. of input lags	4	2	2
No. of estimators	5	10	5
Minimum samples per leaf	5	5	2
Maximum depth	5	5	2
Friedman F rank	3.67	4.33	4.00
**TDNN**			
No. of input lag	4	3	2
No. of hidden nodes	5	1	1
Activation function in hidden node	Logistic	Logistic	Logistic
Total number of weights	31	6	5
Friedman F rank	3.00	3.00	2.33
**Stationary- TDNN**			
No. of input lag	2	2	4
No. of hidden nodes	9	4	3
Activation function in hidden node	Logistic	Logistic	Logistic
Total number of weights	37	17	19
Friedman F rank	1.67	1.33	1.00

This study identified the most effective time-delay neural network with a single hidden layer for both the original and stationary series. By altering the range of input and hidden nodes from 1 to 6 and from 1 to 10, respectively, we have optimized the network performance. The Levenberg-Marquardt back-propagation algorithm has been utilized for training. Detailed specifications of the selected TDNN models can be found in Table 7. The superiority of TDNN models over other machine learning models is evident in the Friedman F ranks presented in the table, indicating it as the prime choice for further decomposition-based improvements. The performance enhancement of TDNN models when utilizing the stationary (first differenced) series as input rather than the original non-stationary series also supports the theory that proper pre-processing techniques (such as differencing and decomposing) of such data can significantly elevate the efficacy of neural network models^40,101^.

#### Fitting of the EMD-TDNN models

EMD has decomposed each price series into independent IMFs and residues through a sifting process. Characteristics and the selected TDNN model specifications of these IMFs and residue are presented in Table 8, whereas the graphical representation of the decomposed series is given in ‘Supplementary Information Fig. S1-S3’. Among the decomposed sub-series, the highest average value has been observed for residue in each case. However, the highest fluctuation has been found for residue in the case of Rapeseed & mustard and Linseed, and for IMF-4 in the case of groundnut. The highest correlation with the original series is observed for IMF-4 in the case of groundnut and for residue in the case of rapeseed & mustard and linseed. Most of the IMFs have exhibited a positive correlation. However, negative correlations are observed for one of the IMFs in the case of groundnut and rapeseed & mustard.

**Table 8:** Characteristics and TDNN model specifications of the IMFs and residue obtained through EMD.

Sub-series	Mean	Standard Deviation	Correlation coefficient	TDNN model
**Groundnut**				
IMF-1	94.76	641.94	0.08	4:3s:1
IMF-2	-23.65	583.43	0.28	3:1s:1
IMF-3	-20.36	251.89	0.23	4:1s:1
IMF-4	-376.05	973.29	0.84	4:4s:1
IMF-5	-82.31	158.80	-0.49	2:6s:1
Residue	4110.50	139.95	-0.24	3:9s:1
**Rapeseed & Mustard**				
IMF-1	7.90	102.22	0.09	3:6s:1
IMF-2	-13.52	258.64	0.28	2:1s:1
IMF-3	-45.30	438.64	0.42	6:9s:1
IMF-4	5.70	44.14	-0.17	4:3s:1
Residue	3741.21	466.99	0.84	8:6s:1
**Linseed**				
IMF-1	-0.11	123.16	0.16	1:9s:1
IMF-2	12.78	164.98	0.25	4:3s:1
IMF-3	-153.78	512.94	0.03	3:4s:1
IMF-4	71.24	313.15	0.14	4:4s:1
Residue	3963.33	977.85	0.84	4:10s:1

#### Fitting of the EEMD-TDNN models

The purpose of ensembling in EEMD is to avoid the problem of mode mixing. ‘Supplementary Information Figure S4-S6’ illustrates the IMFs and residue obtained through EEMD. Like EMD, all the IMFs are obtained from the highest to the lowest frequency. The residue varies slowly around the long-term average. The average fluctuation and correlation patterns of the obtained IMFs are similar to those of EMD and can be observed in Table 9. However, except IMF-3 of linseed, all other IMFs and residue are positively correlated with the original series.

**Table 9:** Characteristics and TDNN model specifications of the IMFs and residue obtained through EEMD.

Sub-series	Mean	Standard Deviation	Correlation coefficient	TDNN model
**Groundnut**				
IMF-1	3.35	352.76	0.26	4:1s:1
IMF-2	9.47	316.04	0.33	3:1s:1
IMF-3	-2.82	238.90	0.45	4:1s:1
IMF-4	-119.60	534.18	0.80	4:4s:1
IMF-5	-34.02	238.84	0.08	4:6s:1
Residue	3848.36	370.79	0.61	4:8s:1
**Rapeseed & Mustard**				
IMF-1	1.74	69.34	0.08	1:7s:1
IMF-2	-1.24	87.60	0.28	4:8s:1
IMF-3	18.11	153.09	0.26	4:8s:1
IMF-4	-4.45	226.18	0.25	3:7s:1
Residue	3667.27	622.42	0.84	3:6s:1
**Linseed**				
IMF-1	-1.97	80.52	0.14	4:9s:1
IMF-2	9.48	134.96	0.25	7:8s:1
IMF-3	-161.69	403.47	-0.05	5:10s:1
IMF-4	-7.64	284.10	0.14	1:8s:1
Residue	4057.08	1068.70	0.82	3:2s:1

#### Fitting of the CEEMD-TDNN models

CEEMD can handle the noise generated by non-negligible residues in the EEMD process. Characteristics and the selected TDNN model specifications of the IMFs and residue obtained through CEEMD are presented in Table 10, whereas the decomposed series is graphically represented in ‘Supplementary Information Fig. S7-S9’. Among the decomposed sub-series, the residue component has consistently displayed the highest average value. Notably, the residue in Rapeseed & Mustard and Linseed, and IMF-1 in groundnut, have exhibited the highest fluctuations among the sub-series. All the IMFs exhibited a positive correlation except for the IMF-4 of groundnut. Among the positive ones, the highest correlation is observed for IMF-5 in the case of groundnut and for residue in the case of rapeseed & mustard, and linseed.

**Table 10:** Characteristics and TDNN model specifications of the IMFs and residue obtained through CEEMD.

Sub-series	Mean	Standard Deviation	Correlation coefficient	TDNN model
**Groundnut**				
IMF-1	99.92	650.08	0.08	3:8s:1
IMF-2	-34.55	581.35	0.26	2:1s:1
IMF-3	-16.54	280.01	0.23	4:10s:1
IMF-4	88.00	362.96	-0.04	3:3s:1
IMF-5	-130.36	565.08	0.68	6:7s:1
Residue	2603.45	541.29	0.62	6:7s:1
**Rapeseed & Mustard**				
IMF-1	3.62	85.39	0.02	3:1s:1
IMF-2	-1.32	151.47	0.18	5:2s:1
IMF-3	-22.08	264.32	0.43	6:10s:1
IMF-4	21.86	190.60	0.09	3:8s:1
Residue	3678.11	582.16	0.84	5:7s:1
**Linseed**				
IMF-1	-1.28	114.36	0.17	6:4s:1
IMF-2	4.75	151.51	0.21	3:1s:1
IMF-3	9.97	166.38	0.20	2:2s:1
IMF-4	-58.05	404.73	0.25	4:7s:1
Residue	3938.07	879.99	0.86	4:8s:1

#### Fitting of the CEEMDAN-TDNN models

CEEMDAN utilizes the same ‘divide and conquer’ framework as the original EMD method. However, by adding finite adaptive white noises, CEEMDAN yields IMFs more stable and closer to a normal distribution than the IMFs obtained through EMD. ‘Supplementary Information Figure S10-S12’ illustrates the IMFs and residue obtained through CEEMDAN. From Table 11, it can be observed that the residue has the highest average value in each case. However, the highest fluctuations have been observed for residue in the case of Rapeseed & mustard and Linseed, as well as for IMF-5 in groundnut. Notably, there is a positive correlation between all IMFs and residue and their original series. The pattern of the highest correlation is the same as CEEMD, i.e., IMF-5 in the case of groundnut and residue in the case of rapeseed & mustard and linseed.

**Table 11:** Characteristics and TDNN model specifications of the IMFs and residue obtained through CEEMDAN.

Sub-series	Mean	Standard Deviation	Correlation coefficient	TDNN model
**Groundnut**				
IMF-1	2.85	352.49	0.26	1:8s:1
IMF-2	-6.36	172.44	0.26	1:10s:1
IMF-3	-4.10	250.59	0.33	2:1s:1
IMF-4	-7.58	201.30	0.40	1:9s:1
IMF-5	-137.66	506.93	0.80	5:3s:1
Residue	3855.74	390.44	0.68	6:9s:1
**Rapeseed & Mustard**				
IMF-1	1.72	69.06	0.08	1:9s:1
IMF-2	-0.17	10.39	0.14	6:6s:1
IMF-3	-1.01	54.70	0.23	6:7s:1
IMF-4	-0.74	105.49	0.28	4:1s:1
Residue	3680.39	621.03	0.97	1:10s:1
**Linseed**				
IMF-1	-1.77	80.53	0.14	4:6s:1
IMF-2	-0.10	16.24	0.16	2:9s:1
IMF-3	1.06	97.15	0.23	7:3s:1
IMF-4	23.04	171.49	0.15	4:4s:1
Residue	3917.30	914.30	0.96	3:3s:1

## Discussion

The comparative results of the time series models under investigation concerning the post-sample RMSE, RRMSE and MAPE values are given in Table 12. The error-based metrics reveal that the values of the forecasted series are closer to the values of the actual price series when obtained using the proposed CEEMDAN-TDNN models. It also reflects an almost uniform order of accuracy, i.e., CEEMDAN-TDNN > CEEMD-TDNN > EEMD-TDNN > EMD-TDNN > Stationary-TDNN > TDNN > RF > GBM > NLSVR > ARIMA for all the three-price series considered. Moreover, it can be noted that as the data series is truly non-linear, non-linear machine learning models such as NLSVR, GBM, RF, TDNN have clear advantages over the model rendering linear forecasts such as ARIMA. Among these machine models, TDNN has shown a comparative edge over the others and hence is considered for further hybridization to obtain more accurate forecasts.

**Table 12:** Comparative assessment of forecasting accuracy.

Model	Groundnut			Rapeseed & mustard			Linseed		
	RMSE	RRMSE	MAPE	RMSE	RRMSE	MAPE	RMSE	RRMSE	MAPE
ARIMA	531.36	11.66	8.20	217.61	4.95	4.51	257.73	5.75	4.99
NLSVR	503.85	10.14	8.26	187.21	1.55	3.44	379.22	5.14	6.28
GBM	491.68	8.35	8.62	154.88	2.94	3.08	412.65	6.45	6.64
RF	488.70	10.62	8.61	191.53	2.25	3.61	411.76	8.71	6.19
TDNN	485.79	10.66	7.45	145.86	3.32	2.99	251.86	5.62	4.88
Stationary-TDNN	480.88	10.55	6.88	91.31	2.08	1.78	213.17	4.76	3.48
EMD-TDNN	375.46	8.24	6.00	79.97	1.82	1.52	196.37	4.38	3.51
EEMD-TDNN	349.98	7.68	5.58	57.76	1.31	1.02	169.20	3.78	3.34
CEEMD-TDNN	310.24	6.81	5.05	59.24	1.35	1.03	151.54	3.38	2.66
CEEMDAN-TDNN	289.24	6.35	3.99	49.89	1.14	0.79	132.82	2.96	2.36

The advantage of using a stationary series as an input to a TDNN model is also substantiated by the superior performance of the stationary-TDNN model over the TDNN model. However, as the underlying non-linear and non-stationary features of commodity prices significantly impact on the robustness of the neural network models^102^, more than mere differencing is needed to handle such multiscale complexity. Hence, the EMD variant-based TDNN models, which can simultaneously deal with non-linear and non-stationary features, have provided far better forecasts^103^. However, one important point that emerged is the potential for using additional explanatory variables for each decomposed component. Each component of the decomposed series (such as trend, seasonality, and high-frequency variations) could potentially be explained by different factors beyond lagged observations. For example, a trend component might be influenced by macroeconomic variables such as inflation or GDP, while short-term fluctuations might be more closely tied to speculative trading or market sentiment. Future work should consider such factors to enhance the interpretability and accuracy of forecasts for agricultural prices. Furthermore, external factors like rainfall or weather conditions, which have a direct impact on agricultural production and price volatility, can also be explored for inclusion.

**Fig. 2:**
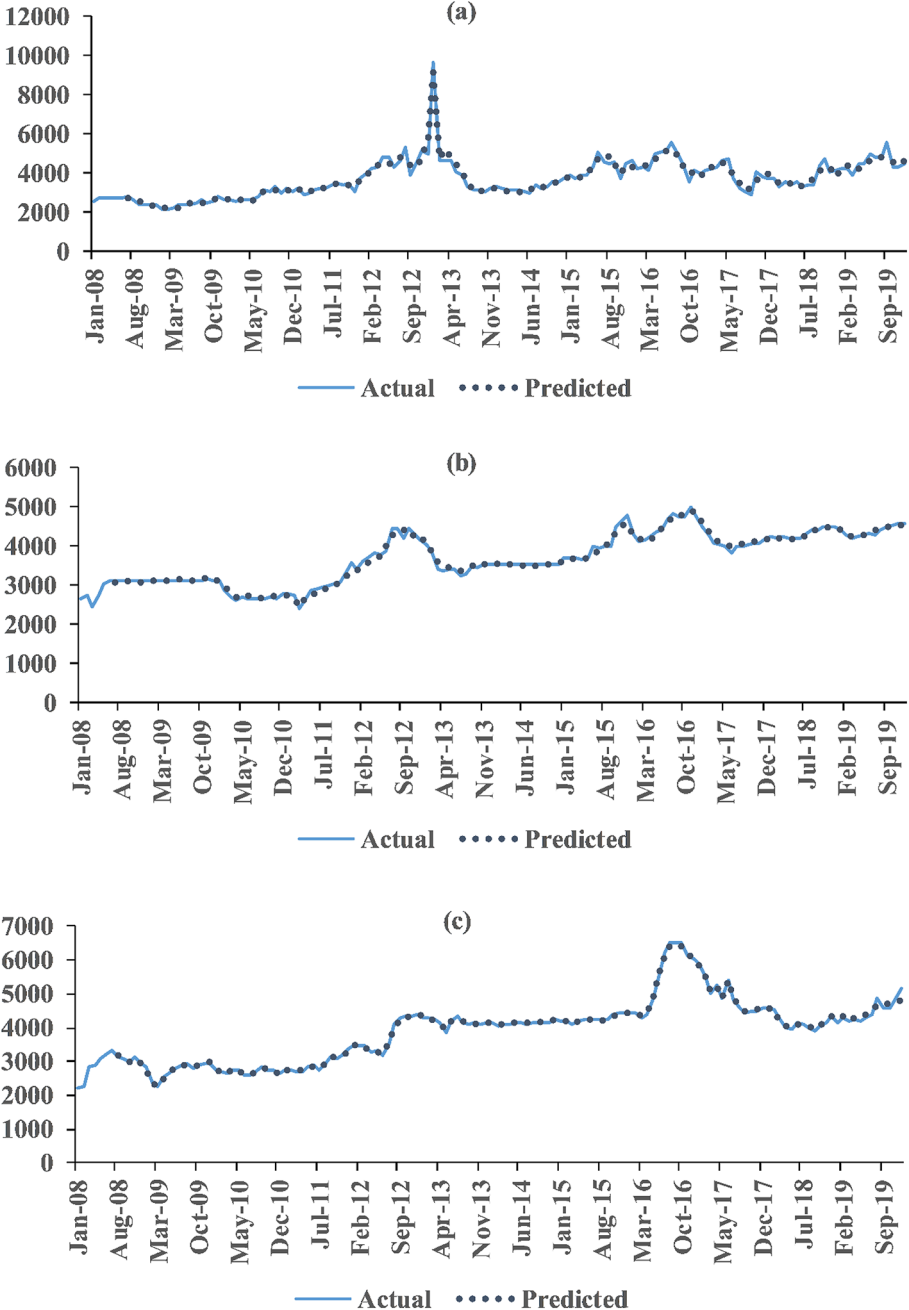
Actual and best-predicted price series (by the CEEMDAN-TDNN hybrid model) of (**a**) groundnut, (**b**) rapeseed & mustard and (**c**) linseed.

It is worth noting that even though EEMD, CEEMD, and CEEMDAN are modifications over EMD, substantial differences in the sub-series characteristics are observed. Consequently, the input series (lagged observations of that sub-series) and the target output (the sub-series) have varied from one decomposition technique to another, allowing the neural networks to capture patterns in significantly different ways^83^, which certainly affects the model performance. The gradual improvements in the accuracy of EEMD over EMD and CEEMD over EEMD have been evident, mainly due to the ensembling algorithm and the use of complementary white noise pair, respectively. Zhang et al.^104^ have also observed such incremental performance of EMD-variants for neural network forecasting of groundwater depth prediction. Finally, the remarkable improvements in the accuracy of CEEMDAN as opposed to almost all of its counterparts and marginal improvement over CEEMD can be attributed to the extra noise coefficient vector $\:{\upomega\:}$, which controls the noise level at each stage of decomposition. In this technique, the signal is adaptively decomposed and noise adaptive processing is carried out on individual sub-series components, enabling better adaptation to the non-linear and non-smooth characteristics of the signal, which significantly enhance the decomposition accuracy and stability. The efficiency of the CEEMDAN-based hybrid models, due to its unique feature extraction technique, was also observed in the studies of Gao et al.^105^ for predicting nitrogen content in citrus leaves, Bennia et al.^106^ for minimizing the impact of additive noises on non-invasive biomedical signals, Gyamerah and Owusu^107^ for improving weather prediction amidst extreme climate change in Africa, etc. Time plots of actual vs. predicted series employing CEEMDAN-TDNN models are presented in Fig. 2.

However, as indicated earlier, several researchers have suggested that the conventional error-based metrics may not be suitable for evaluating non-linear models, since a linear model can outperform the non-linear ones even when the true data-generating process is non-linear^87,108^. A non-linear model can generate comparatively more variation in the forecast values than a linear model. Hence, errors with a larger magnitude are likely to be unduly penalized. Table 13 provides the post-sample percentage of forecasts of correct signs.

**Table 13:** Post-sample percentage of forecasts of correct sign.

Model	Groundnut	Rapeseed & mustard	Linseed
ARIMA	66.67	66.67	66.67
NLSVR	50.00	66.67	33.33
GBM	41.67	66.67	41.67
RF	50.00	58.33	91.67
TDNN	41.67	41.67	66.67
Stationary-TDNN	66.67	66.67	66.67
EMD-TDNN	66.67	83.33	75.00
EEMD-TDNN	66.67	91.67	75.00
CEEMD-TDNN	75.00	91.67	83.33
CEEMDAN-TDNN	75.00	91.67	83.33

**Fig. 3:**
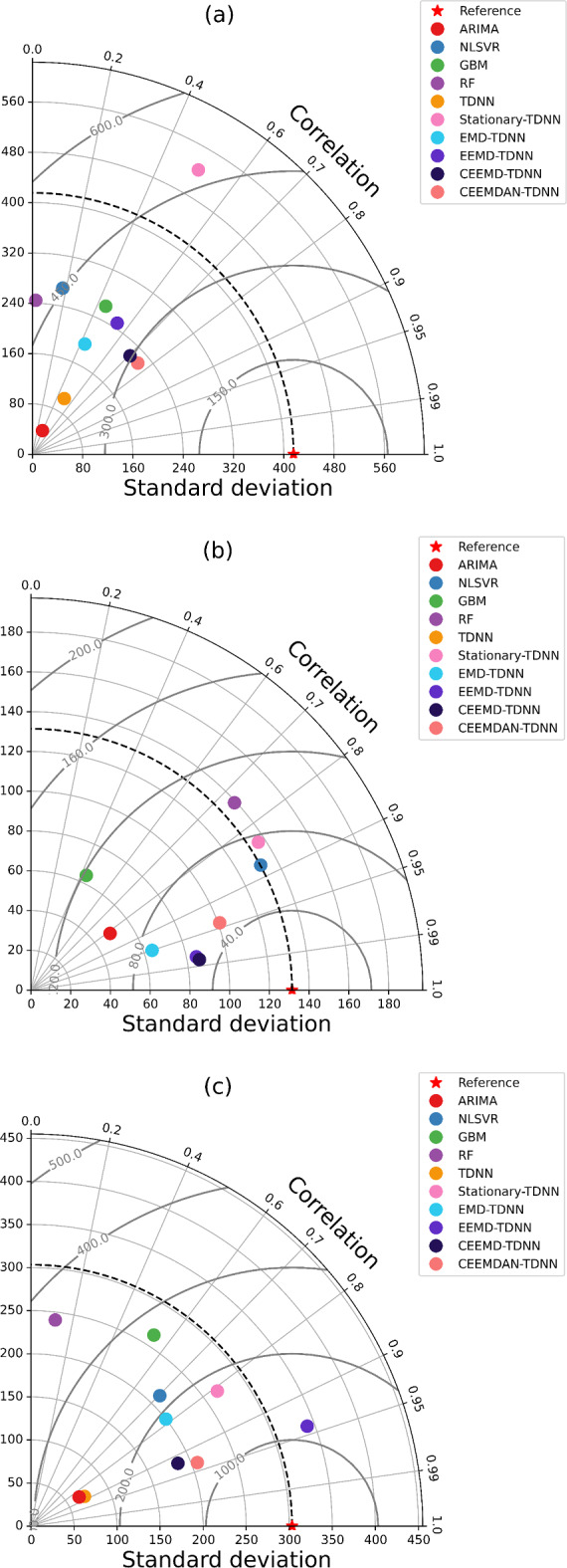
Taylor diagram of the forecasting models for (**a**) groundnut, (**b**) rapeseed & mustard and (**c**) linseed price series.

The linear ARIMA model has performed equally or even better than the non-linear machine learning models. At this juncture, the impact of the inherent non-linearity and non-stationarity on the performance of the machine learning models is distinctly more realized in terms of turning point prediction. Among the EMD-variant based hybrids, comparable performance is observed in the EMD-TDNN and EEMD-TDNN models. The CEEMD-TDNN and CEEMDAN-TDNN models have also exhibited the equal ability to forecast the change direction. Thus, the comprehensive assessment of the forecasting models indicates that the relative forecasting performance also crucially relies on the evaluation metrics. The superiority of the forecasting accuracy of the CEEMDAN-TDNN hybrid over the other competing models is determined by both the DM test and the Friedman test. The results of the DM and Friedman test (‘Supplementary Information Table S1 and S2’) along with the Taylor diagram confirm that the CEEMDAN-TDNN has outperformed other benchmark models in forecasting accuracy across all series (Fig. 3).

## Conclusions

This study has evaluated the suitability of the CEEMDAN-TDNN hybrid model for non-linear, non-stationary agricultural price series forecasting in comparison with the other three major EMD variants (EMD, EEMD and CEEMD) and the benchmark (ARIMA, NLSVR, GBM, RF and TDNN) models using monthly wholesale prices of major oilseed crops in India. Outcomes from this investigation reflect that the CEEMDAN-TDNN models have provided uniformly better results than all other forecasting models regarding RMSE, RRMSE and MAPE values. However, in the case of turning point prediction, the CEEMD-TDNN model has exhibited the same ability as the CEEMDAN-TDNN model.

One important avenue for future research is incorporating additional explanatory variables tailored to each decomposed component. For instance, macroeconomic factors like inflation or export trends could be used to explain long-term trends, while short-term fluctuations could be modeled based on speculative or market-related variables. Weather variables such as rainfall, which directly affect agricultural production and, consequently, price movements, can be critical for improving the forecasting accuracy of price volatility. A richer set of explanatory variables will make the models more robust and provide better insights into the factors driving price dynamics.

The proposed hybrid model can be applied to agricultural data and similar time series data such as stock market, weather, pollution data, etc. Future research will explore implementing other machine learning or deep learning models to enhance efficiency further. Additionally, exploring the use of the Improved CEEMDAN (ICEEMDAN) method in place of CEEMDAN to assess potential improvements in results would be of interest.

## Electronic supplementary material

Below is the link to the electronic supplementary material.

Supplementary Material 1

## Data Availability

The data supporting this study’s findings are available from the corresponding author upon reasonable request.
