# Supplementary material for: Hybrid modeling approaches for agricultural commodity prices using CEEMDAN and time delay neural networks
Source: Sci Rep. 2024 Nov 4;14:26639. doi: 10.1038/s41598-024-74503-4 (PMC11535273; doi:10.1038/s41598-024-74503-4)
Supplement: Supplementary file 1 — Supplementary Material 1 [file 41598_2024_74503_MOESM1_ESM.docx]

**S1** **Tests of Significance for Equal Forecasting Ability**

The Diebold-Mariano (DM) test is utilised to assess the predictive performance of two competing models. The hypothesis being tested is that the two forecasts are equally accurate, resulting in a loss differential, $d_{(t)}=f\left( e_{1(t)} \right)-f\left( e_{2(t)} \right); t=1,2,\ldots,n$, with an expected value of zero. In this equation, $e_{1(t)}$ and $e_{2(t)}$ represent the forecast error series generated by the two models, and f(.) denotes a loss function. The DM test statistic can be expressed as follows:

| $DM=\frac{\bar{d}}{\sqrt{\hat{v}(\bar{d})}}$ | (1) |
| --- | --- |

where n represents the size of the predictions, $\bar{d}=\frac{1}{n}\sum_{i=1}^{n} d_{(t)}$, $\hat{v}\left( \bar{d} \right)=\frac{1}{n}[\gamma_{0}+2\sum_{k=1}^{h-1} \hat{\gamma}_{k}]$. $\hat{\gamma}_{k}=\frac{1}{n}[\sum_{i=k+1}^{n} \left( d_{\left( t \right)}-\bar{d} \right)(d_{\left( t-k \right)}-\bar{d})]$ are the sample mean of the loss differential, estimate of variance of the mean using h step forecasts and estimate of $k^{\mathrm{th}}$ autocovariance of $d_{(t)}$, respectively.

Further, the Friedman test is utilised for reliable assessment of accuracy across various models. It is a non-parametric test employed for variance analysis based on rankings of two-way classified data. The test serves as a multiple comparison tool to rank various forecasting models according to significant differences in their results. The null hypothesis of the test posits equal forecasting performance among all models. The test procedure commences with the assignment of ranks based on the predicted results of each model individually. The procedure of the test starts with assigning ranks according to the predicted results of all the models separately. If the rank of a model is $m^{\mathrm{th}}$according to $j^{\mathrm{th}}$ accuracy measures and it is denoted by $r_{j}^{m}$ (j = 1, 2, …, n and m = 1, 2, …, k), the average rank of that model can be obtained by: $R_{m}=\frac{1}{n}\sum_{j=1}^{n} r_{j}^{m}$ , where n and k are the number of accuracy measures and forecasting models considered, respectively. The Friedman test statistic is determined using the following formula:

| $F=\frac{12n}{k\left( k+1 \right)}\left[ \sum_{m=1}^{k} R_{m}^{2}-\frac{k{(k+1)}^{2}}{4} \right]$ | (2) |
| --- | --- |

In case of significant differences, a post-hoc procedure can be used to characterise these variations. By applying a post-hoc test, a p value can be obtained to assess the degree of rejection for each hypothesis. The test statistic z for comparing the $p^{\mathrm{th}}$ and $q^{\mathrm{th}}$ models is calculated as:

| $z=\left( R_{p}-R_{q} \right)/{\sqrt{\frac{k\left( k+1 \right)}{6n}}}$ | (3) |
| --- | --- |

where $R_{p}$ and $R_{q}$ represent the average rankings determined by the Friedman test for the models under comparison. The z-value is utilised to determine the corresponding probability (p-value) from the normal distribution N(0, 1) table in each instance, and this value is then compared against the designated level of significance α.


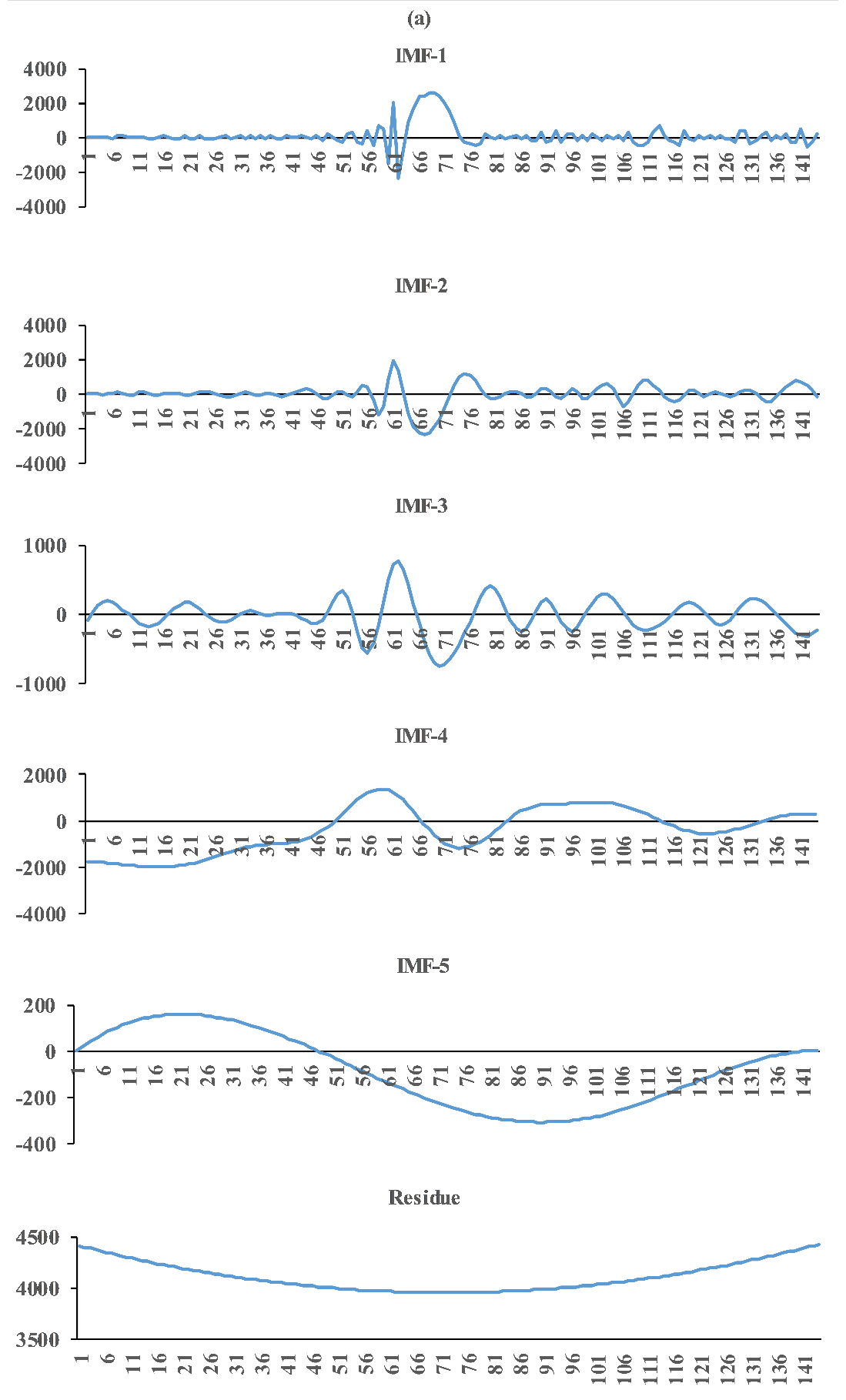


**Fig. S1.** IMFs and residue obtained through EMD for groundnut price series.


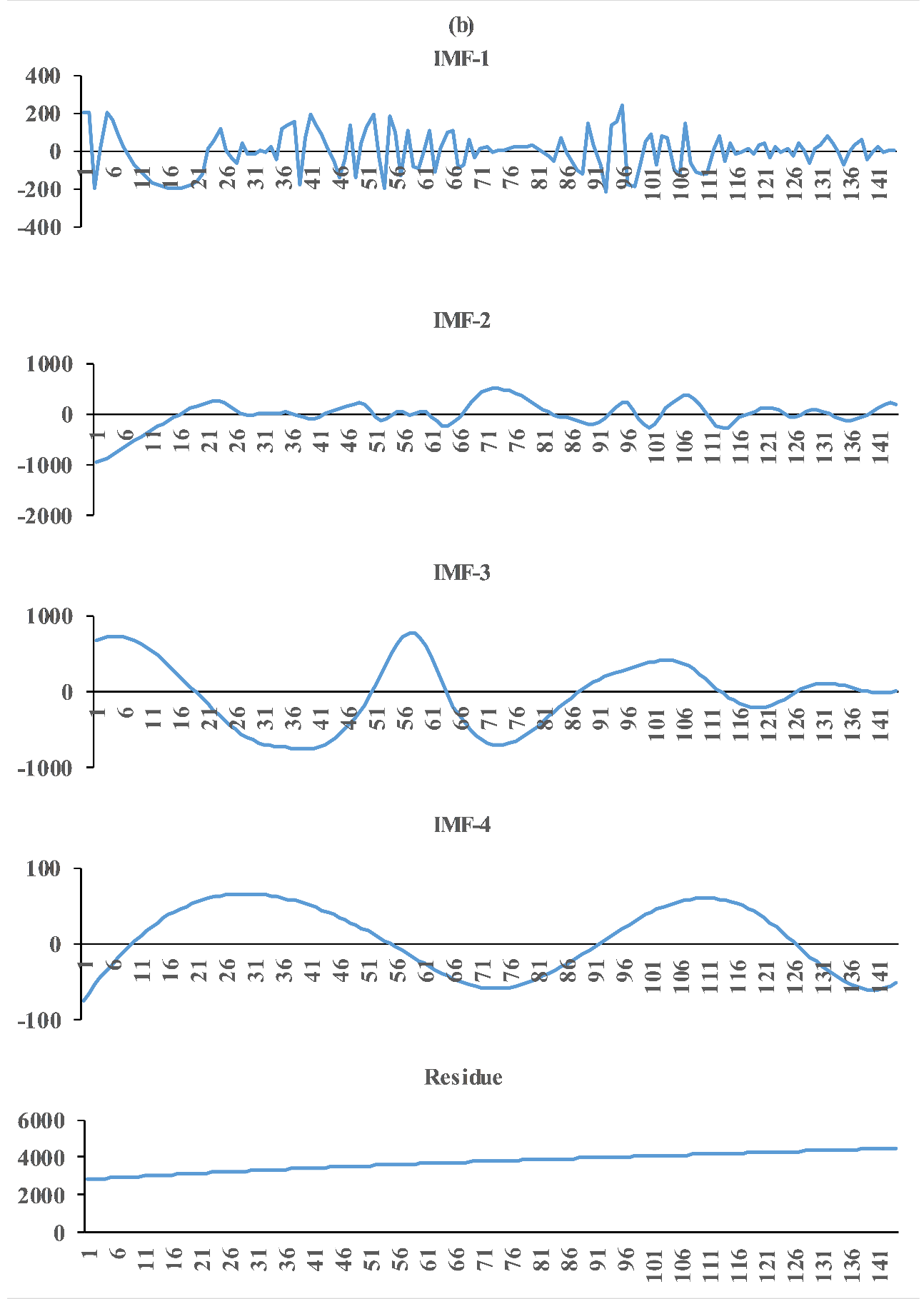


**Fig. S2.** IMFs and residue obtained through EMD for rapeseed & mustard price series.


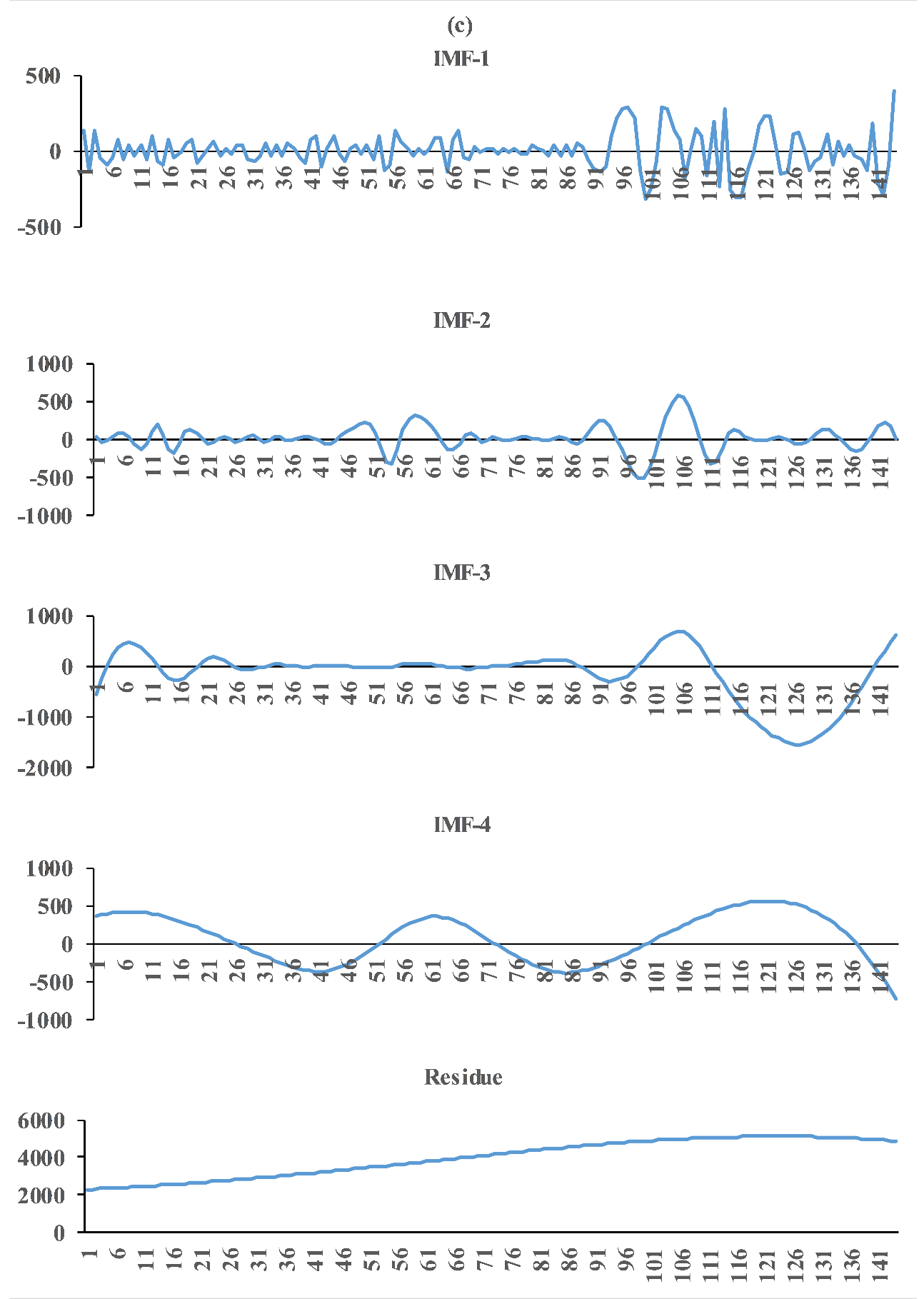


**Fig. S3.** IMFs and residue obtained through EMD for linseed price series.


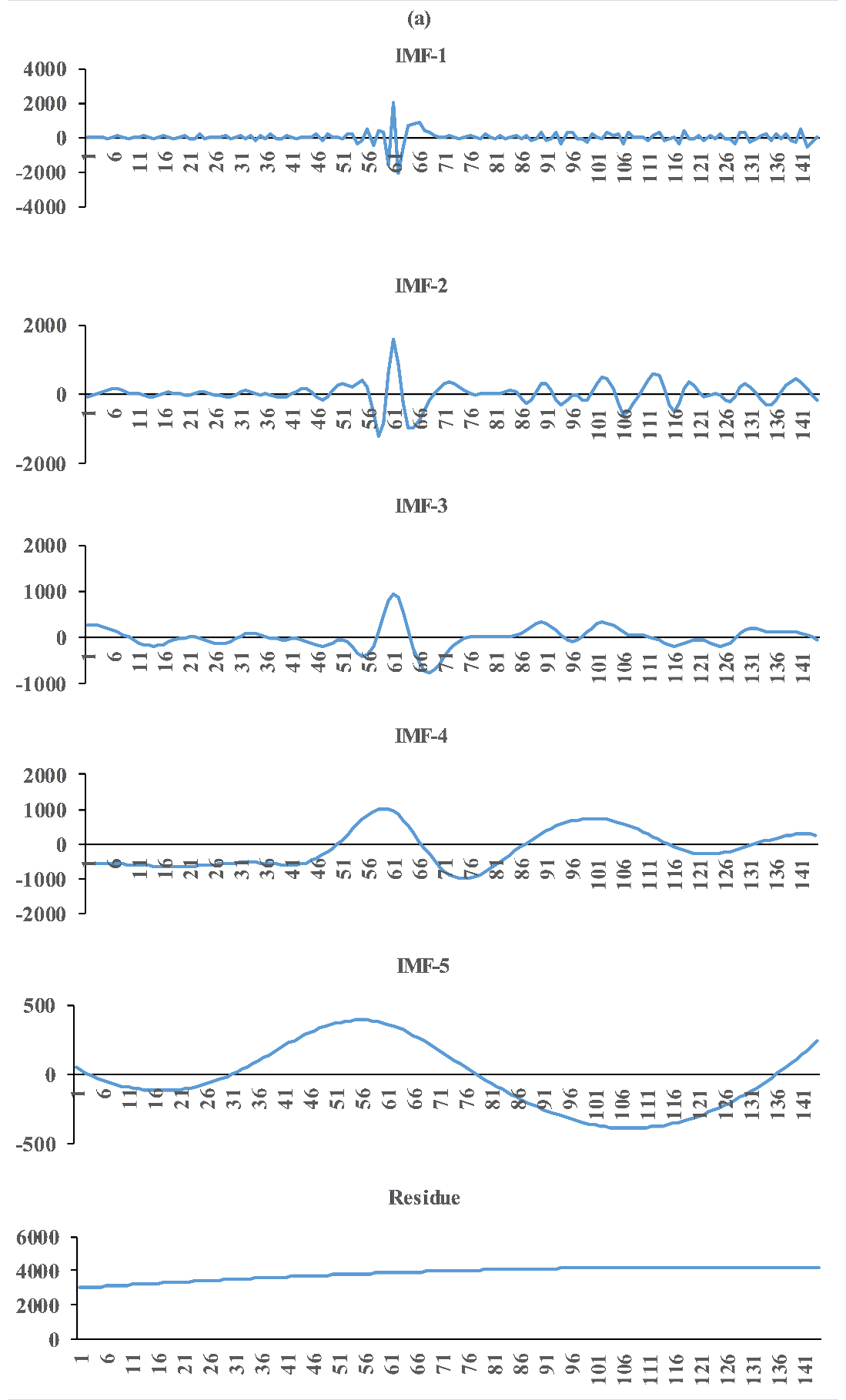


**Fig. S4.** IMFs and residue obtained through EEMD for groundnut price series.


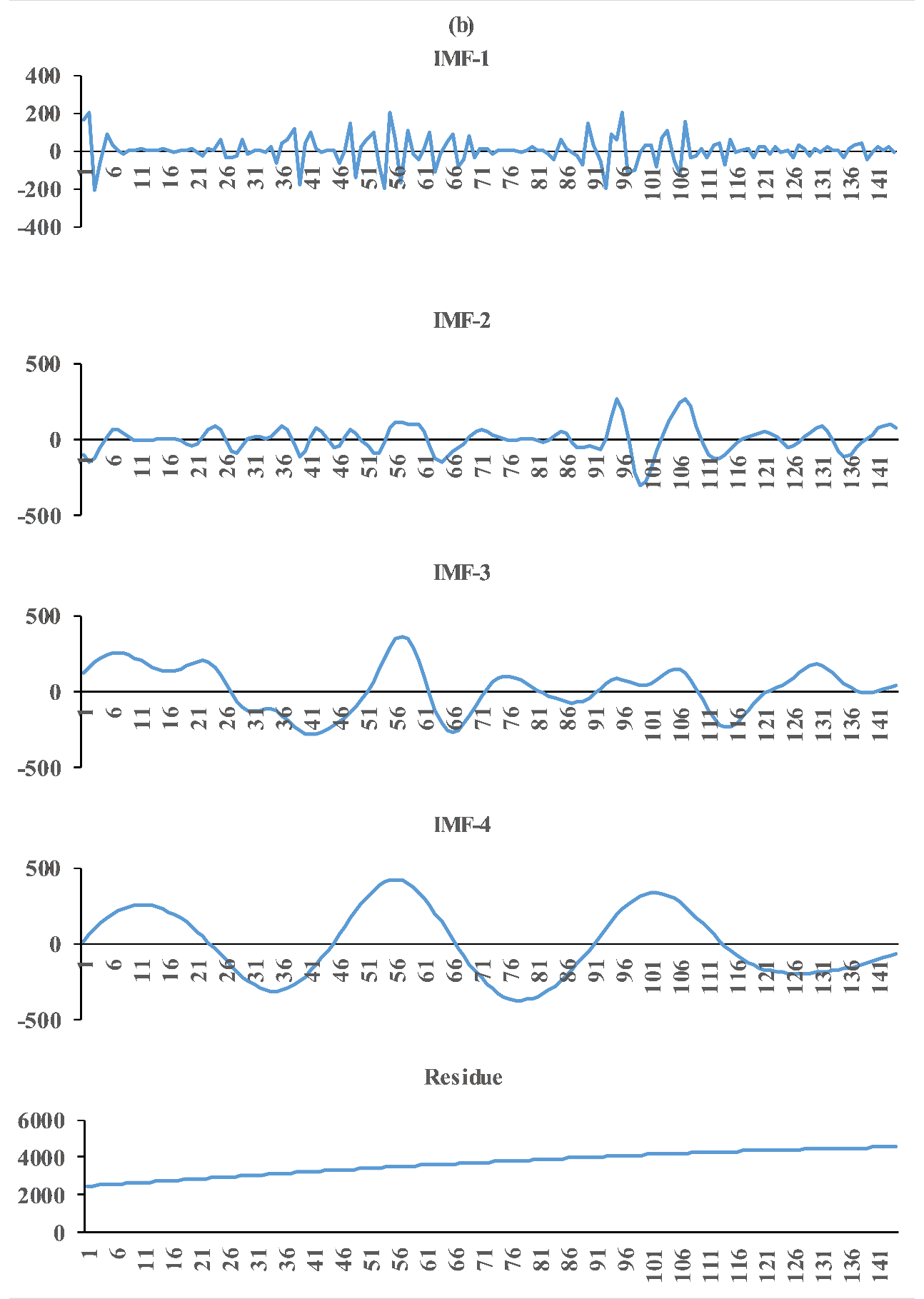


**Fig. S5.** IMFs and residue obtained through EEMD for rapeseed & mustard price series.


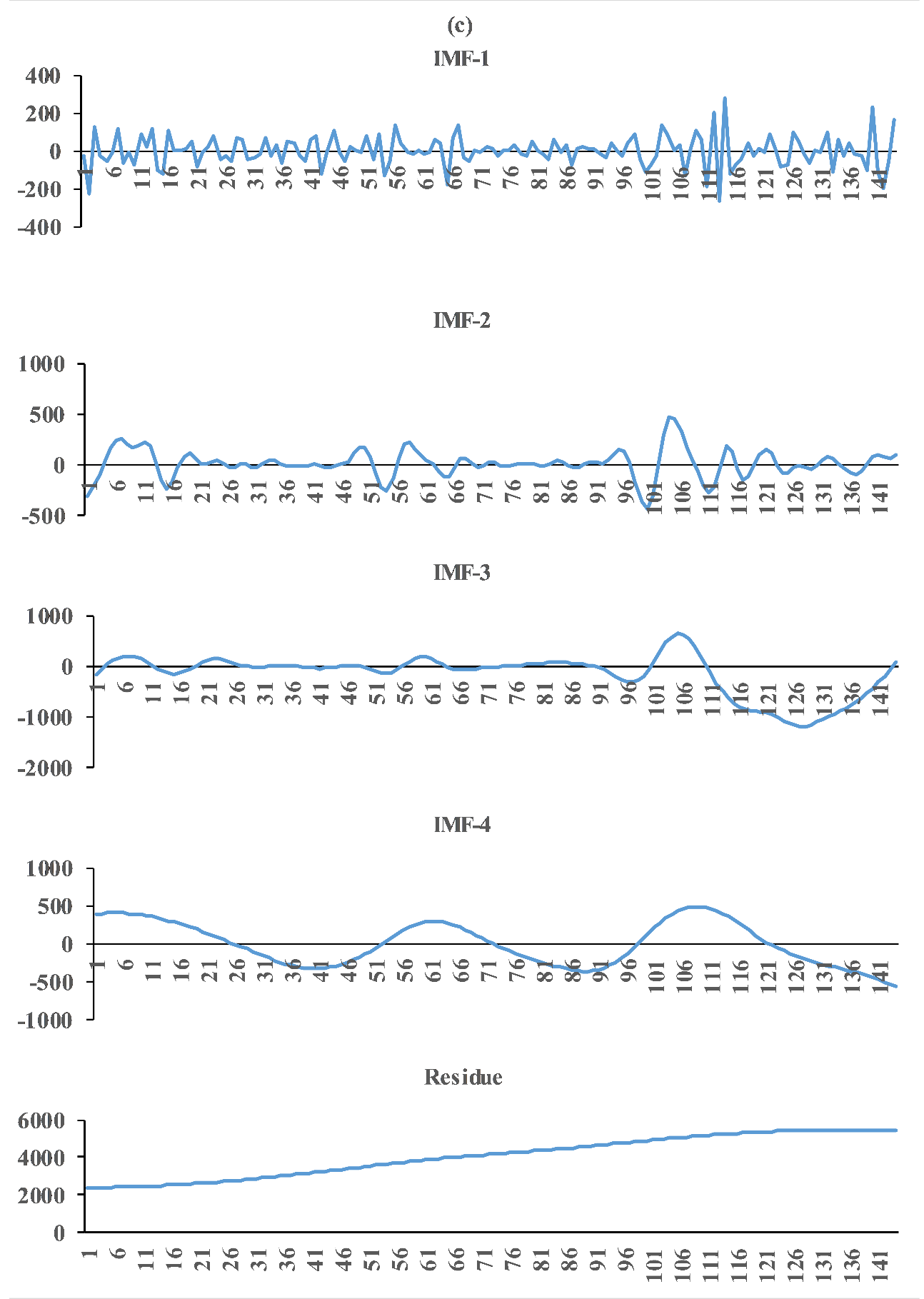


**Fig. S6.** IMFs and residue obtained through EEMD for linseed price series.


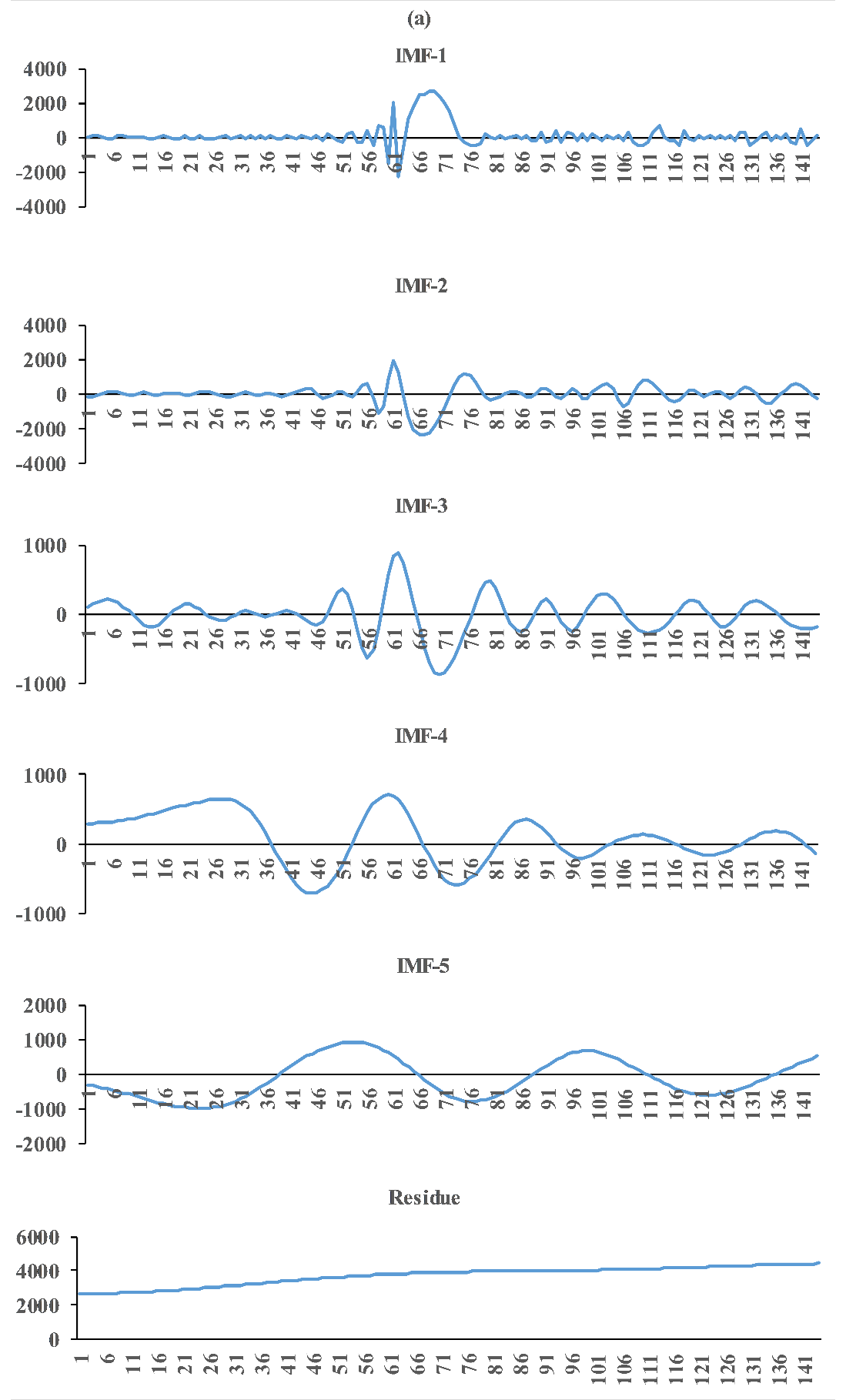


**Fig. S7.** IMFs and residue obtained through CEEMD for groundnut price series.


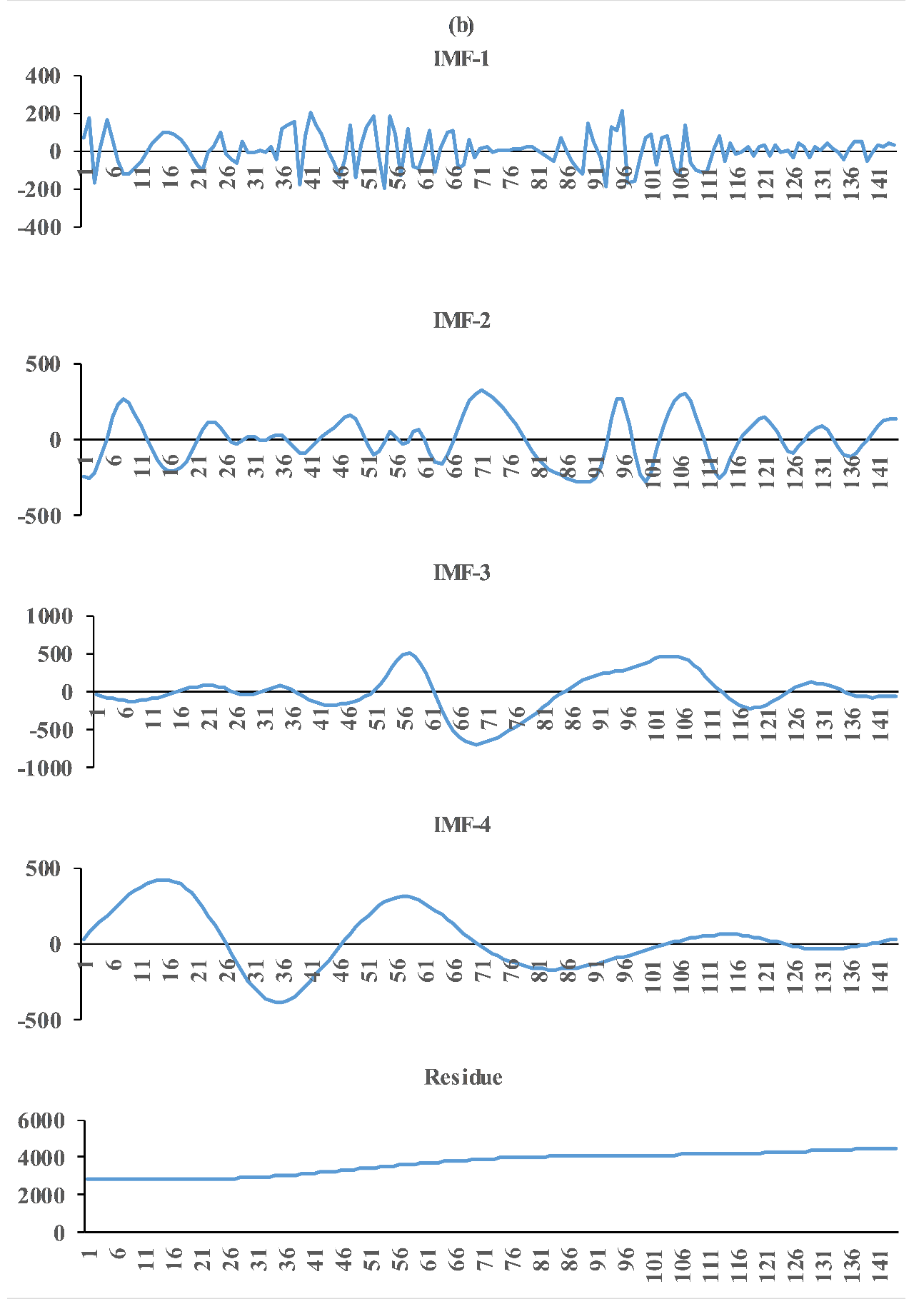


**Fig. S8.** IMFs and residue obtained through CEEMD for rapeseed & mustard price series.


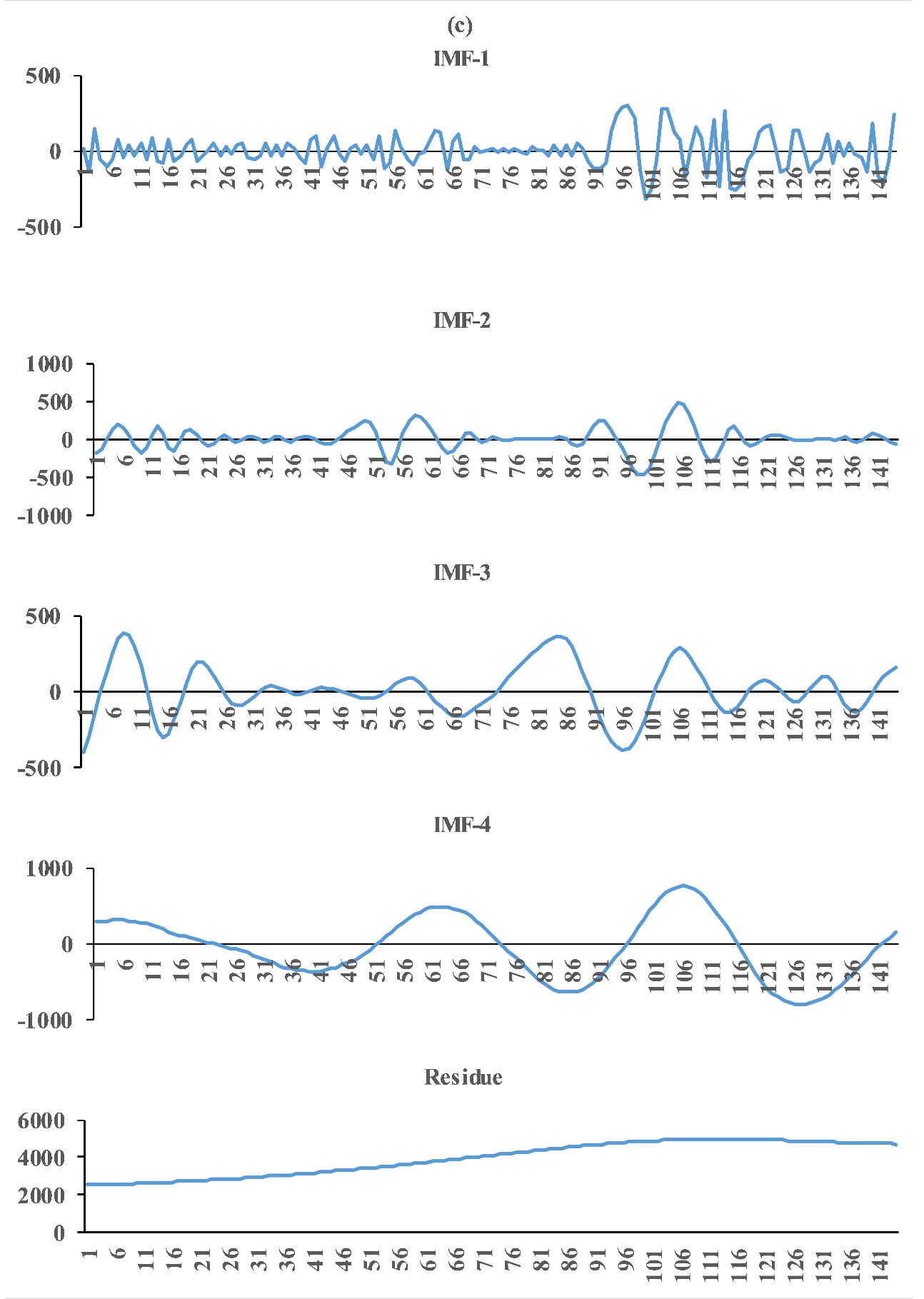


**Fig. S9.** IMFs and residue obtained through CEEMD for linseed price series.


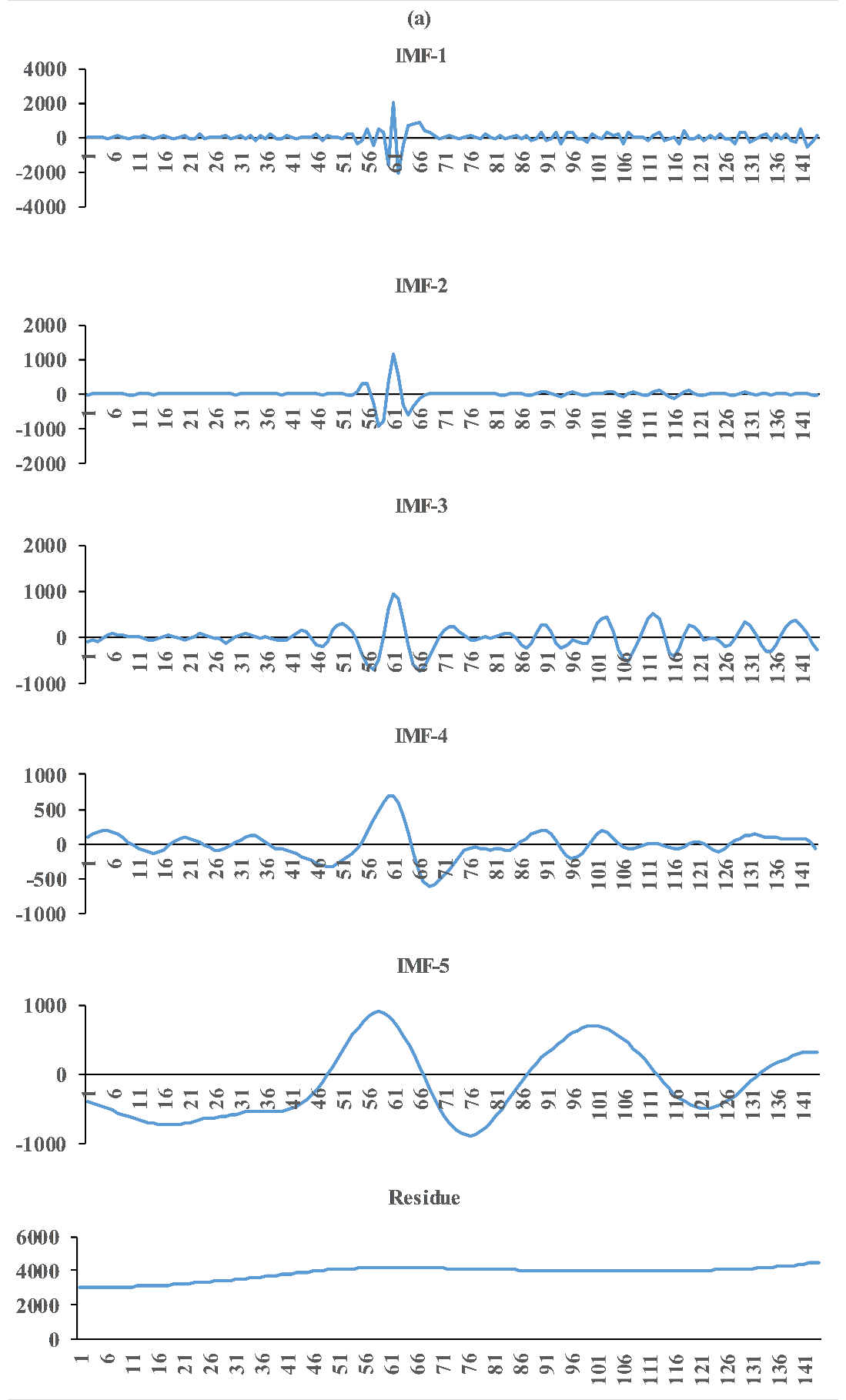


**Fig. S10.** IMFs and residue obtained through CEEMDAN for groundnut price series.


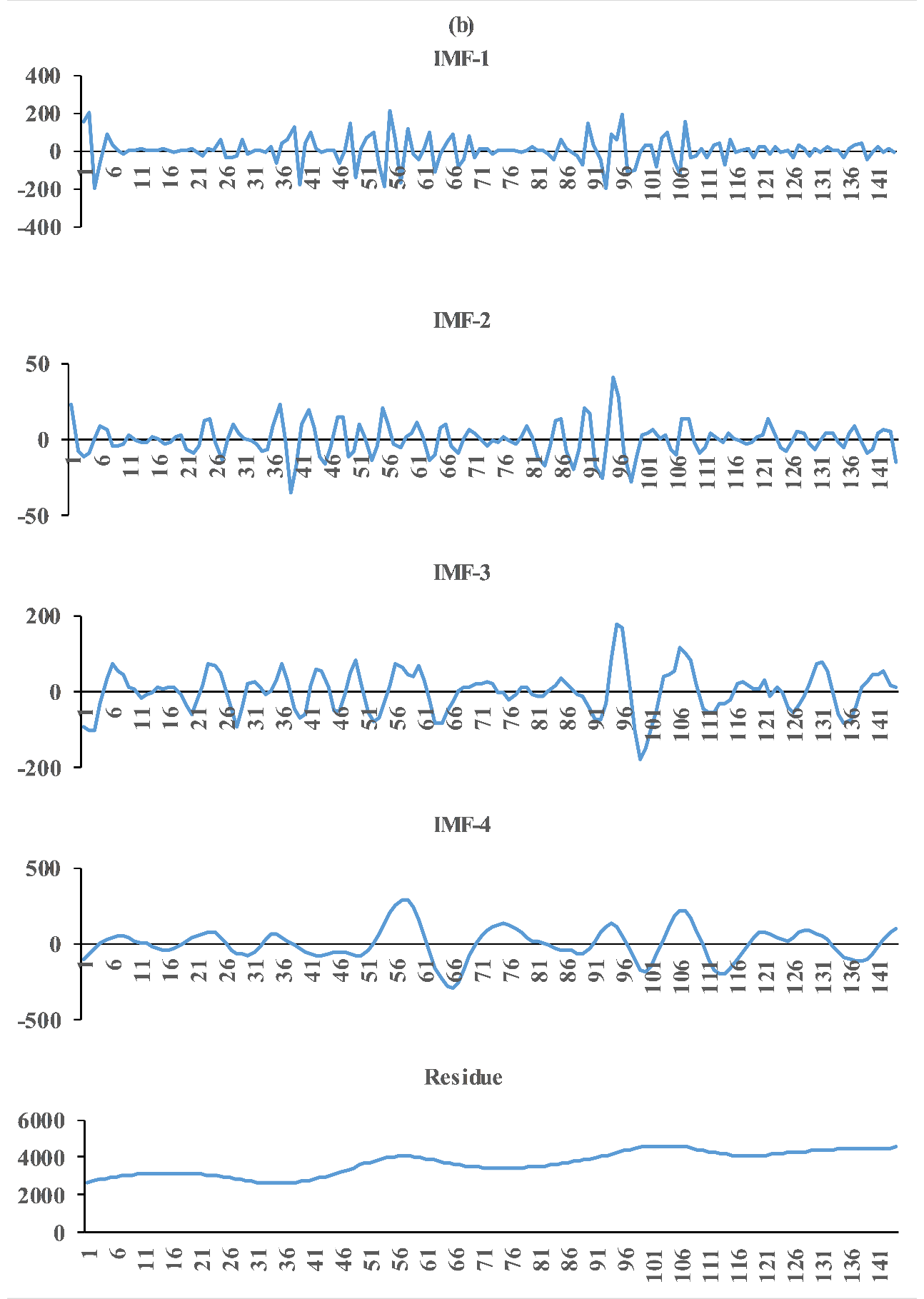


**Fig. S11.** IMFs and residue obtained through CEEMDAN for rapeseed & mustard price series.


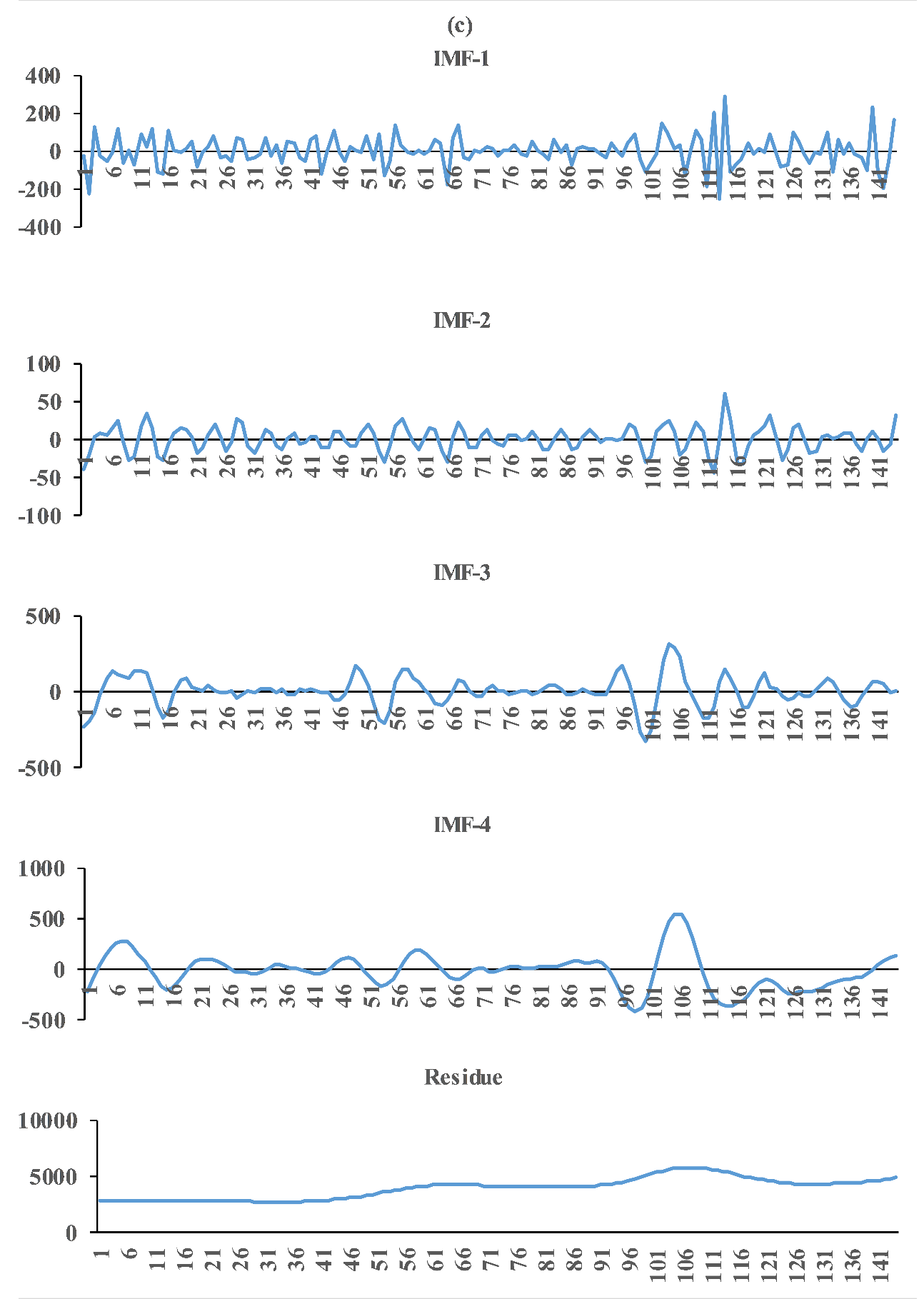

**Fig. S12.** IMFs and residue obtained through CEEMDAN for linseed price series.

**Table S1.** Results of the Diebold–Mariano test.

| **Tested models** | **Benchmark models** | | | | | | | | | | | |
| --- | --- | --- | --- | --- | --- | --- | --- | --- | --- | --- | --- | --- |
|  | DM statistics and p value (in bracket) | | | | | | | | | | | |
|  | Groundnut | | | | | | | | | | | |
|  | **ARIMA** | **NLSVR** | **GBM** | **RF** | **TDNN** | **Stationary-TDNN** | **EMD-TDNN** | **EEMD-TDNN** | **CEEMD-TDNN** |  |  |  |
| **NLSVR** | 0.85 (0.41) |  |  |  |  |  |  |  |  |  |  |  |
| **GBM** | 1.38 (0.20) | 1.82 (0.10) |  |  |  |  |  |  |  |  |  |  |
| **RF** | 0.41 (0.69) | -0.42 (0.68) | -1.91 (0.08) |  |  |  |  |  |  |  |  |  |
| **TDNN** | 2.12 (0.06) | -0.32 (0.75) | -1.04 (0.32) | -0.02 (0.99) |  |  |  |  |  |  |  |  |
| **Stationary-TDNN** | 0.29 (0.78) | -0.16 (0.88) | -0.71 (0.49) | 0.03 (0.97) | 0.03 (0.97) |  |  |  |  |  |  |  |
| **EMD-TDNN** | 1.62 (0.13) | 1.70 (0.12) | 0.13 (0.90) | 1.54 (0.15) | 1.32 (0.21) | 0.66 (0.52) |  |  |  |  |  |  |
| **EEMD-TDNN** | 1.43 (0.18) | 1.53 (0.15) | 0.81 (0.43) | 1.95 (0.07) | 1.16 (0.27) | 0.81 (0.43) | 17.79 (<0.01) |  |  |  |  |  |
| **CEEMD-TDNN** | 1.74 (0.11) | 2.47 (0.03) | 1.69 (0.12) | 2.86 (0.02) | 1.49 (0.16) | 1.17 (0.26) | 1.07 (0.30) | 0.70 (0.50) |  |  |  |  |
| **CEEMDAN-TDNN** | 2.17 (0.05) | 3.36 (0.01) | 2.50 (0.02) | 2.81 (0.01) | 1.93 (0.08) | 1.18 (0.26) | 1.69 (0.12) | 1.00 (0.34) | 0.62 (0.54) |  |  |  |
| Rapeseed & mustard | | | | | | | | | | | |  |
| **NLSVR** | 3.87 (<0.01) |  |  |  |  |  |  |  |  | |  |  |
| **GBM** | 3.28 (0.01) | -2.10 (0.06) |  |  |  |  |  |  |  | |  |  |
| **RF** | 3.22 (0.01) | -1.73 (0.11) | 1.01 (0.33) |  |  |  |  |  |  | |  |  |
| **TDNN** | 2.32 (0.04) | -3.07 (0.01) | -0.96 (0.35) | -1.60 (0.13) |  |  |  |  |  | |  |  |
| **Stationary-TDNN** | 3.25 (0.01) | -1.47 (0.17) | 1.46 (0.17) | 0.37 (0.71) | 2.52 (0.03) |  |  |  |  | |  |  |
| **EMD-TDNN** | 3.29 (0.01) | -0.58 (0.58) | 1.60 (0.13) | 0.74 (0.47) | 3.36 (0.01) | 0.54 (0.60) |  |  |  | |  |  |
| **EEMD-TDNN** | 3.65 (<0.01) | 0.57 (0.58) | 2.12 (0.06) | 1.64 (0.13) | 3.79 (<0.01) | 1.67 (0.12) | 55.30 (<0.01) |  |  | |  |  |
| **CEEMD-TDNN** | 3.59 (<0.01) | 0.46 (0.65) | 2.06 (0.06) | 1.58 (0.14) | 3.79 (<0.01) | 1.58 (0.14) | 2.74 (0.02) | -0.58 (0.57) |  | |  |  |
| **CEEMDAN-TDNN** | 3.95 (<0.01) | 1.20 (0.25) | 2.39 (0.03) | 2.16 (0.05) | 3.78 (<0.01) | 1.88 (0.09) | 1.84 (0.09) | 0.66 (0.52) | 0.73 (0.48) | |  |  |
| Linseed | | | | | | | | | | | | |
| **NLSVR** | 0.63 (0.53) |  |  |  |  |  |  |  |  |  |  |  |
| **GBM** | -0.52 (0.61) | -1.46 (0.17) |  |  |  |  |  |  |  |  |  |  |
| **RF** | -1.49 (0.16) | -1.92 (0.08) | -1.63 (0.13) |  |  |  |  |  |  |  |  |  |
| **TDNN** | 2.29 (0.04) | -0.51 (0.62) | 0.62 (0.55) | 1.53 (0.15) |  |  |  |  |  |  |  |  |
| **Stationary-TDNN** | 0.95 (0.36) | 1.41 (0.19) | 1.71 (0.12) | 1.90 (0.09) | 0.84 (0.42) |  |  |  |  |  |  |  |
| **EMD-TDNN** | 2.46 (0.03) | 0.64 (0.53) | 1.43 (0.18) | 1.97 (0.07) | 2.26 (0.04) | 0.30 (0.77) |  |  |  |  |  |  |
| **EEMD-TDNN** | 1.59 (0.14) | 0.98 (0.34) | 1.61 (0.14) | 1.86 (0.09) | 1.52 (0.16) | 0.73 (0.48) | 23.95 (<0.01) |  |  |  |  |  |
| **CEEMD-TDNN** | 3.20 (0.01) | 1.96 (0.08) | 2.06 (0.06) | 2.11 (0.06) | 3.23 (0.01) | 1.64 (0.13) | 1.30 (0.22) | 0.40 (0.70) |  |  |  |  |
| **CEEMDAN-TDNN** | 3.74 (<0.01) | 2.01 (0.07) | 2.23 (0.05) | 2.21 (0.05) | 3.78 (<0.01) | 1.68 (0.12) | 2.05 (0.06) | 0.91 (0.38) | 1.24 (0.24) |  |  |  |

**Table S2.** Results of the Friedman test.

| **Tested models** | **Benchmark models** | | | | | | | | | | | |
| --- | --- | --- | --- | --- | --- | --- | --- | --- | --- | --- | --- | --- |
|  | Friedman test statistics and p value (in bracket) | | | | | | | | | | | |
|  | Groundnut (Overall test statistic value of model rankings being different = 23.29, p value <0.01) | | | | | | | | | | | |
|  | **ARIMA** | **NLSVR** | **GBM** | **RF** | **TDNN** | **Stationary-TDNN** | **EMD-TDNN** | **EEMD-TDNN** | **CEEMD-TDNN** |  |  |  |
| **NLSVR** | 0.54 (0.59) |  |  |  |  |  |  |  |  |  |  |  |
| **GBM** | 0.54 (0.59) | 0.00 (1.00) |  |  |  |  |  |  |  |  |  |  |
| **RF** | 0.40 (0.69) | -0.13 (0.89) | -0.13 (0.89) |  |  |  |  |  |  |  |  |  |
| **TDNN** | 0.81 (0.42) | 0.27 (0.79) | 0.27 (0.79) | 0.40 (0.69) |  |  |  |  |  |  |  |  |
| **Stationary-TDNN** | 1.35 (0.18) | 0.81 (0.42) | 0.81 (0.42) | 0.94 (0.35) | 0.54 (0.59) |  |  |  |  |  |  |  |
| **EMD-TDNN** | 2.02 (0.04) | 1.48 (0.14) | 1.48 (0.14) | 1.62 (0.11) | 1.21 (0.22) | 0.67 (0.50) |  |  |  |  |  |  |
| **EEMD-TDNN** | 2.43 (0.02) | 1.89 (0.06) | 1.89 (0.06) | 2.02 (0.04) | 1.62 (0.11) | 1.08 (0.28) | 0.40 (0.69) |  |  |  |  |  |
| **CEEMD-TDNN** | 2.83 (<0.01) | 2.29 (0.02) | 2.29 (0.02) | 2.43 (0.02) | 2.02 (0.04) | 1.48 (0.14) | 0.81 (0.42) | 0.40 (0.69) |  |  |  |  |
| **CEEMDAN-TDNN** | 3.24 (<0.01) | 2.70 (0.01) | 2.70 (0.01) | 2.83 (<0.01) | 2.43 (0.02) | 1.89 (0.06) | 1.21 (0.22) | 0.81 (0.42) | 0.40 (0.69) |  |  |  |
| Rapeseed & mustard (Overall test statistic value of model rankings being different = 24.67, p value <0.01) | | | | | | | | | | | |  |
| **NLSVR** | 1.35 (0.18) |  |  |  |  |  |  |  |  | |  |  |
| **GBM** | 1.08 (0.28) | -0.27 (0.79) |  |  |  |  |  |  |  | |  |  |
| **RF** | 0.67 (0.5) | -0.67 (0.50) | -0.40 (0.69) |  |  |  |  |  |  | |  |  |
| **TDNN** | 1.21 (0.22) | -0.13 (0.89) | 0.13 (0.89) | 0.54 (0.59) |  |  |  |  |  | |  |  |
| **Stationary-TDNN** | 1.89 (0.06) | 0.54 (0.59) | 0.81 (0.42) | 1.21 (0.22) | 0.67 (0.50) |  |  |  |  | |  |  |
| **EMD-TDNN** | 2.29 (0.02) | 0.94 (0.35) | 1.21 (0.22) | 1.62 (0.11) | 1.08 (0.28) | 0.40 (0.69) |  |  |  | |  |  |
| **EEMD-TDNN** | 3.24 (<0.01) | 1.89 (0.06) | 2.16 (0.03) | 2.56 (0.01) | 2.02 (0.04) | 1.35 (0.18) | 0.94 (0.35) |  |  | |  |  |
| **CEEMD-TDNN** | 2.83 (<0.01) | 1.48 (0.14) | 1.75 (0.08) | 2.16 (0.03) | 1.62 (0.11) | 0.94 (0.35) | 0.54 (0.59) | -0.40 (0.69) |  | |  |  |
| **CEEMDAN-TDNN** | 3.64 (<0.01) | 2.29 (0.02) | 2.56 (0.01) | 2.97 (<0.01) | 2.43 (0.02) | 1.75 (0.08) | 1.35 (0.18) | 0.40 (0.69) | 0.81 (0.42) | |  |  |
| Linseed (Overall test statistic value of model rankings being different = 25.91, p value <0.01) | | | | | | | | | | | | |
| **NLSVR** | -0.13 (0.89) |  |  |  |  |  |  |  |  |  |  |  |
| **GBM** | -0.94 (0.35) | -0.81 (0.42) |  |  |  |  |  |  |  |  |  |  |
| **RF** | -0.67 (0.50) | -0.54 (0.59) | 0.27 (0.79) |  |  |  |  |  |  |  |  |  |
| **TDNN** | 0.40 (0.69) | 0.54 (0.59) | 1.35 (0.18) | 1.08 (0.28) |  |  |  |  |  |  |  |  |
| **Stationary-TDNN** | 1.08 (0.28) | 1.21 (0.22) | 2.02 (0.04) | 1.75 (0.08) | 0.67 (0.50) |  |  |  |  |  |  |  |
| **EMD-TDNN** | 1.21 (0.22) | 1.35 (0.18) | 2.16 (0.03) | 1.89 (0.06) | 0.81 (0.42) | 0.13 (0.89) |  |  |  |  |  |  |
| **EEMD-TDNN** | 1.75 (0.08) | 1.89 (0.06) | 2.70 (0.01) | 2.43 (0.02) | 1.35 (0.18) | 0.67 (0.50) | 0.54 (0.59) |  |  |  |  |  |
| **CEEMD-TDNN** | 2.16 (0.03) | 2.29 (0.02) | 3.10 (<0.01) | 2.83 (<0.01) | 1.75 (0.08) | 1.08 (0.28) | 0.94 (0.35) | 0.40 (0.69) |  |  |  |  |
| **CEEMDAN-TDNN** | 2.56 (0.01) | 2.70 (0.01) | 3.51 (<0.01) | 3.24 (<0.01) | 2.16 (0.03) | 1.48 (0.14) | 1.35 (0.18) | 0.81 (0.42) | 0.40 (0.69) |  |  |  |
